# Supplementary material for: Dome patterns in pelagic size spectra reveal strong trophic cascades
Source: Nat Commun. 2019 Sep 27;10:4396. doi: 10.1038/s41467-019-12289-0 (PMC6764997; doi:10.1038/s41467-019-12289-0)
Supplement: Supplementary file 1 — Supplementary Information [file 41467_2019_12289_MOESM1_ESM.pdf]

Supplementary Information

for

Dome patterns in pelagic size spectra reveal strong trophic cascades

Axel G. Rossberg, Ursula Gaedke, and Pavel Kratina

August 21, 2019

Contents

Supplementary Note 1 Definition of the non-linear Species Size-Spectrum Model 2

Supplementary Note 2 Model parameterization 6

Supplementary Note 3 Model Simulations 9

Supplementary Note 4 Empirical size spectra and fitted modulated lines 10

Supplementary Note 5 Regressions of empirical size-spectrum characteristics *vs* nutrient enrichment 19

Supplementary Note 6 The linear SSSM and its responses to size-specific pressures 19

6.1 Formulation of the linear SSSM 19
6.2 Fourier transforms 20
6.3 Linear response theory for the SSSM 20
6.4 Demonstration of the recipe on a simple example 23

Supplementary Note 7 Analytic theory for the formation of dome patterns 27

7.1 Implications of a changing size-spectrum slope 27
7.2 Implications of overall biomass increase 28
7.3 Changes in the direction of propagation of trophic cascades in the linear SSSM 34
7.4 Conclusions 35

Supplementary Note 1: Definition of the non-linear Species Size-Spectrum Model

The non-linear SSSM builds on and generalises the linear SSSM that we presented in an earlier study [1]; below we refer to this study as 'CAT', base on the first few words on the paper's title ("A complete analytic theory..."), and provide pointers to relevant passages and equations for easy reference. In the present context, a 'linear' model means one where deviations of the size spectrum from an ideal power law are assumed to be so small that a linear approximation for the dynamics of these deviations is valid. By contrast, the non-linear SSSM admits arbitrary deviations of the size spectrum from an ideal power law. Its derivation follows that of the linear SSSM up to the point where the linearization is mathematically carried out. This derivation (extending over 24 pages) does not need to be repeated here. It leads to Equation [59] of CAT, an approximate formula for the momentary linear growth rate $\Lambda(m_*)$ of populations of species with maturation body mass m_* (there written as "$\langle V_{m_*}|L_{m_*}W_{m_*}\rangle$"):

$$\Lambda(m_*) = \int_0^\infty \left[(\alpha f(m)h - k)m^n - m\,\mu_p(m)\right] W_{m_*}(m)dm. \tag{1}$$

In this integral, m denotes the body mass of individuals (independent of their species identity); $f(m)$ denotes their feeding level (a number between 0 and 1 quantifying the degree of satiation); α is the assimilation efficiency; h and k are coefficients scaling maximum intake rate and respiration rate, respectively; the factor m^n describes the allometric scaling of these rates with body mass; $\mu_p(m)$ is the predation mortality at body size m; and $W_{m_*}(m)$ represents the size structure of populations with maturation body size m_*, normalised to unit population biomass ($\int mW_{m_*}(m)dm = 1$). Parameter values and formulae for computing the functions $f(m)$, $\mu_p(m)$, and $W_{m_*}(m)$ are given in Supplementary Table 1. The computation consists of several steps, which are illustrated in Supplementary Figure 1.

The derivation of Eq. (1) makes use of an approximation that, except for variations in maturation body size m_*, all individuals of a given size m are ecologically equivalent. Further, it takes into account (i) that an accurate unstructured model for the dynamics of a structured populations can be obtained by tracking gains and losses in the total reproductive value [4] of that population [5, 3], and (ii) that in simple size-structured models, such as the one underlying Eq. (1), the reproductive value of individuals is proportional to their body mass [6, Section 6.4.3] and so total reproductive value proportional to population biomass. Hence, the term in brackets in Eq. (1) evaluates biomass gains (somatic growth and reproduction) and losses (mortality) by individuals of size m. These are then summed up for populations of species with a given maturation body size m_* and corresponding population structure $W_{m_*}(m)$.

The non-linear SSSM describes the dynamics of the density $B(m_*)$ of community biomass along the linear m_*-axis. That is, for any maturation body mass interval $[m_*, m_* + \Delta m_*]$ that is not too wide (e.g. $\Delta m_* \ll m_*$), the total biomass (or spatial biomass density) of all species in

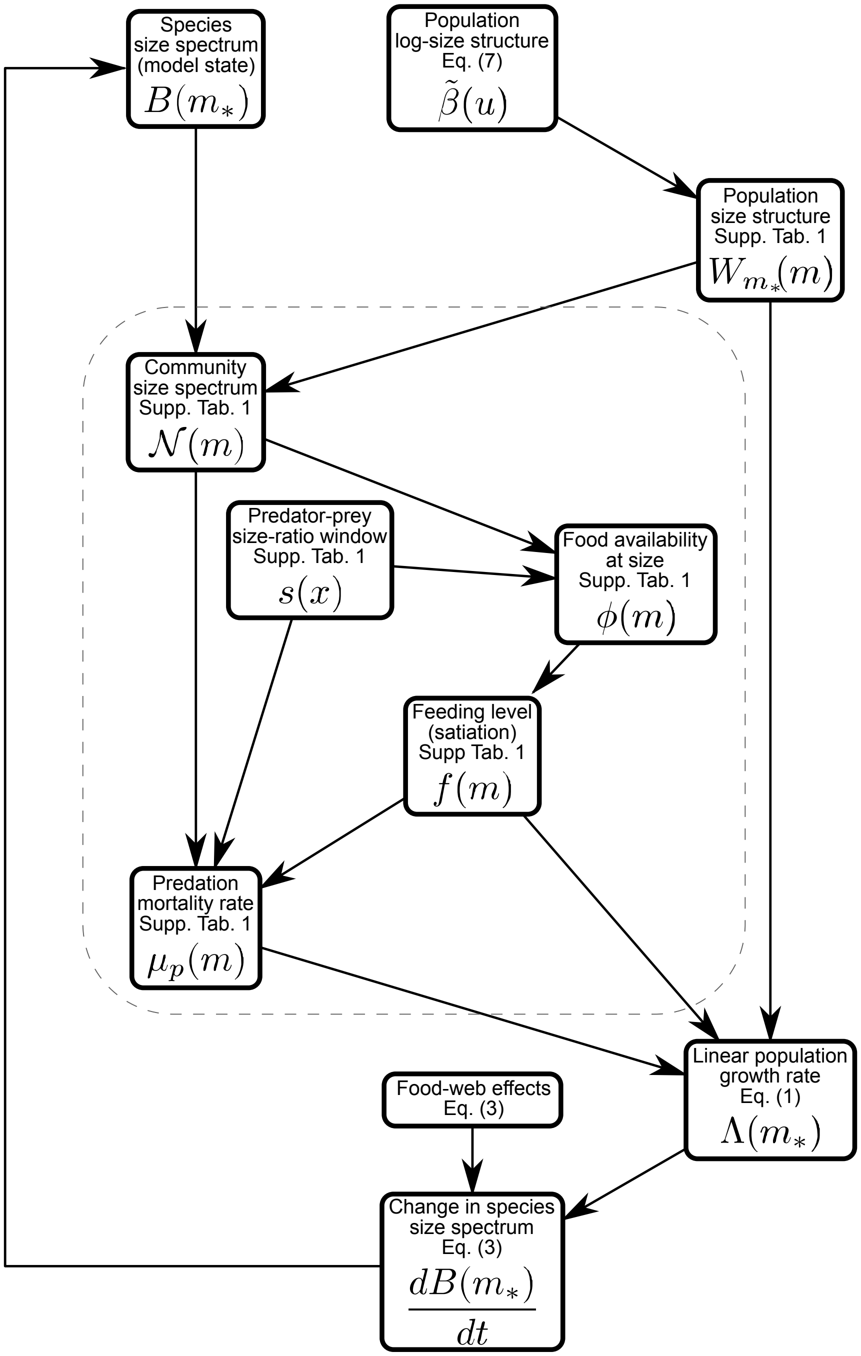

Supplementary Figure 1: Dependencies between size-dependent quantities in the definition of the SSSM. Arrows indicate how each quantity enters the formula for other quantities. The dependencies enclosed by the dashed line follow the model of Ref. [2]. The other dependencies follow from the application of the quasi-neutral approximation [3] to this model, derived in CAT.

a community that have maturation body mass in this interval is $B(m_*)\Delta m_*$. From $B(m_*)$, one can compute the density of biomass along the $(\ln m_*)$-axis as $m_* B(m_*)$, the density of individuals along the linear m-axis $\mathcal{N}(m)$ (Supplementary Table 1), the density of biomass along the linear body mass axis (the so-called normalised *community size spectrum*) as $m\mathcal{N}(m)$, and the density of biomass along the $(\ln m)$-axis, computed as $m^2\mathcal{N}(m)$, which is the representation used in the main text.

In principle, dynamics in the model are given by the simple equation

$$\frac{dB(m_*)}{dt} = \Lambda(m_*)B(m_*). \tag{2}$$

However, in order to suppress artefacts resulting from modelling a food-web with distinct species by a continuum of species characterised by size alone, CAT (Section 6.4) adds a term that damps fluctuations on small scales along the $(\ln m_*)$-axis, resulting in the modified dynamic equation

$$\begin{aligned}\frac{dB(m_*)}{dt} =& \Lambda(m_*)B(m_*)+ \\ & \rho m_*^{n-1}\left\{ B(m_*)\exp\left[\frac{\sigma_r^2(\lambda-2)^2}{2}\right] - \frac{\int_0^\infty B(m'_*)\exp\left[-\frac{\log^2\left(\frac{m'_*}{m_*}\right)}{2\sigma_r^2}\right]dm'_*}{m_*\sqrt{2\pi}\sigma_r}\right\}\end{aligned} \tag{3}$$

(see Supplementary Table 1 for the values of λ, σ_r and ρ). The added correction appears complex here because we write it for a linear m_* scale. When going over to logarithmic size variables, it simplifies considerably, see Eq. (26) below. The role of the additional term is to suppress a model artefact that arises from model simplifications (called coarse graining in the literature) that amount to the assumption that the strength of feeding interactions depends only on the body sizes of consumers and resources. When this dependence is continuous in both resource and consumer body sizes (as it is here), this assumption implies that species of very similar size have very similar consumers and resources and therefore compete strongly with each other. This strong competition among species of similar sizes means that the biomass in a narrow species size class can arbitrarily grow (or decline) while the biomass of a narrow neighbouring species size class declines (or grows), without otherwise affecting community dynamics, as long as the total biomass in the two species size classes remains constant. Minute irregular deviations from this neutral competition will lead to irregular increases and decreases of biomasses of species of similar size, resulting in a ragged species size spectrum that is smooth

only when averaged over sufficiently wide intervals on the log-species-size axis. In reality food webs are more complex because traits other than body mass matter as well [7]. Two species of similar size can therefore have very different sets of consumers and resources, and then do not directly compete with each other. Frequent competitive exclusion of species of similar size would only occur for (hypothetical) communities that are excessively over-saturated with species. Competitive exclusion would then lead to extinction of species until a (near) natural species richness is reached. Natural communities are in such a state: whenever competitive exclusion would happen, it has (mostly) happened already. In natural communities competition between species of similar size is therefore smaller than expected from the simplifying assumption that body-size alone controls feeding interactions.

The correction term puts this right by damping modulations of small wavelength along the species size spectrum. It is constructed such that dynamics on scales much longer than σ_r along the ($\ln m_*$)-axis are not affected. The parameter ρ controls the strength of the suppression of artificial competitive exclusion amongst species of similar size in the model. The particular form of the correction and constraints on the parameters were derived in CAT based on heuristic ecological considerations. A systematic analytical derivation of the correction from first principles would require a better understanding of food-web structure and dynamic than we currently have.

The transition from the linear to the non-linear SSSM permits introduction of a boundary condition representing the upper cutoff of the species size spectrum, above which abundances decline to zero. This is done by simply fixing

$$B(m_*) = 0 \qquad\qquad \text{for } m_* > m_{*\text{max}}, \tag{4}$$

with $m_{*\text{max}}$ representing the maturation body mass of the largest species present in the community.

The lower boundary condition is implemented in the model by fixing

$$B(m_*) = x\tilde{B}_{\text{tot}} m_*^{1-\lambda} \qquad\qquad \text{for } m_* < m_{*\text{min}}. \tag{5}$$

The constant $\tilde{B}_{\text{tot}}$ is chosen such that $B(m_*) = \tilde{B}_{\text{tot}} m_*^{1-\lambda}$ corresponds to the ideal equilibrium power-law species size spectrum in the so called *oligotrophic regime* of the model, where the feeding level $f(m) = f_0$ is the same for all body sizes m, with some constant $0 < f_0 < 1$ (CAT, Section 2.1 and p. 457). It is given by

$$\tilde{B}_{\text{tot}} = \frac{\tilde{\mathcal{N}}}{\int_{-\infty}^{\infty} \mathrm{e}^{(\lambda-2)u} \tilde{\beta}(u) du}, \tag{6a}$$

where

$$\tilde{\mathcal{N}} = \frac{k}{\sqrt{2\pi}\gamma\sigma_s\left\{\beta^{n-1}\mathrm{e}^{(n-1)^2\sigma_s^2/2} - \left(\alpha - \frac{k}{h}\right)\beta^{\lambda-2}\mathrm{e}^{(\lambda-2)^2\sigma_s^2/2}\right\}} \tag{6b}$$

is the corresponding power-law coefficient for the community size spectrum. The dimensionless scale factor x in Eq. (5) can be varied to represent variations in trophic state. To achieve a smooth transition between m_*-ranges of fixed $B(m_*)$ for $m_* < m_{*\mathrm{min}}$ and dynamic $B(m_*)$ for $m_{*\mathrm{min}} \leq m_* \leq m_{*\mathrm{max}}$, a further damping term $-0.5\mathrm{g}^{1-n}\mathrm{yr}^{-1}\, m_*^{n-1}[B(m_*) - y\tilde{B}_{\mathrm{tot}}m_*^{1-\lambda}]/[1 + (m_*\beta^{-1}m_{*\mathrm{min}}^{-1})^{1/2}]$ is added on the right-hand side of Eq. (3), following CAT (Section 8.2). It affects dynamics only for $m_* \lesssim \beta m_{*\mathrm{min}}$, with β representing the typical predator-prey mass ratio.

Supplementary Note 2: Model parameterization

Because of several simplifications of empirical laws invoked in the construction of the SSSM, one cannot expect there to be a "correct" set of values for the model's life-history and physiological parameters that could be determined through direct measurements. Instead, the question we ask here when discussing the model's parameterization is just whether the values we did choose are broadly in line with the numerical ranges observed for corresponding empirical parameters. Our choices of model parameters (Supplementary Table 1) are based on those by [2] and CAT (Section 8.1), with some modifications required to make the model more representative of pelagic communities and to reproduce the empirical characteristics of dome formation. When there was a choice, we preferred values representative of zooplankton, as zooplankters are intermediate within the range of characteristics relevant for pelagic size spectra.

Compared to [2], the allometric coefficient for metabolic losses was reduced by a factor 4 to $2.5\mathrm{g}^{1-n}\mathrm{yr}^{-1}$. The empirical value for this parameter depends on body architecture [8]. Our choice comes closer to that found for copepods, while the original value was rather representative of fish [8, Fig. 1b]. The parameter specifying the preferred predator-prey mass ratio was raised to $\beta = 500$ from 100. Both values are compatible with the empirical range of values for aquatic organisms in general [9, Fig. 3] and for planktonic predators in particular [10, Fig. 3]. In line with the increase in β, the width of the predator-prey mass ratio window on the $\ln(m)$ scale was increased to $\sigma_s = 1.5$ from 1. Assimilation efficiency was reduced from 0.6 to $\alpha = 0.3$, a typical value for zooplankton [11, Tab. 1]. The allometric exponent for search&attack rates was increased from $p = 0.8$ to $p = 0.9$ to better reproduce observed values around -1.10 for the slope $-1-p+n$ of normalised size spectra at low nutrient levels [12, 13]. The coefficient γ for search&attack rates was adjusted from $1.6 \times 10^4\ \mathrm{g}^{1-q}\mathrm{m}^{-3}\mathrm{yr}^{-1}$ [2, Eq. 16] to $10^5\ \mathrm{g}^{1-q}\mathrm{m}^{-3}\mathrm{yr}^{-1}$ to account for our quantification of biomass in terms of carbon rather than wet weight and to

reproduce observed biomass density in pelagic systems (Fig. 3a). The value of the coefficient of the damping term in Eq. (3) is constrained only by a dimensional analysis. It has the same dimensions as the coefficients of the rates of food-intake h and metabolic losses r. Combining these, one obtains a corresponding coefficient for the maximum rate of food assimilation minus losses, $\alpha h - r$. In our parameterization, this evaluates to $23\,\mathrm{g}^{1-n}\mathrm{yr}^{-1}$. Our choice $\rho = 10\,\mathrm{g}^{1-n}\mathrm{yr}^{-1}$ is consistent with the expectation that ρ is of similar magnitude. The heuristic argument of CAT to constrain the width of the size range of damping, σ_r, suggests it should be of the magnitude of the typical distance on the $(\ln m_*)$-axis between the main predators or prey of a species. Assuming that a typical consumer has approximately three prey species making a major contribution the diet [14, 6] (and fewer "main" predators), and noting that we set the width of the predator-prey mass ratio window to $\sigma_s = 1.5$, our choice $\sigma_r = 0.5 = \sigma_s/3$ is consistent with these considerations.

Finally, the simple approximation derived in CAT (Eq. [114]) for the distribution $\tilde{\beta}(u)$ of a species' biomass over the logarithmic body size axis $u = \ln(m/m_*)$ was replaced by

$$\tilde{\beta}(u) = \frac{\eta(1-n)}{\eta + (1-n)\eta^n - \eta x_0^{1-n}} \begin{cases} 0 & \text{if } \exp(u) < x_0, \\ \mathrm{e}^{u(1-n)} & \text{if } x_0 \le \exp(u) < 1, \\ \dfrac{\mathrm{e}^{(2-n)u/(1-n)}\eta^n(\mathrm{e}^{-u}\eta^n - \mathrm{e}^{-nu}\eta)^{1/(1-n)}}{(\eta^n - \eta)^{1/(1-n)}[(\mathrm{e}^u\eta)^n - \mathrm{e}^u\eta]} & \text{if } 1 \le \exp(u) < \eta^{-1}, \\ 0 & \text{if } \eta^{-1} \le \exp(u). \end{cases} \tag{7}$$

This expression describes a steady-state size distribution of individuals that, after being born at size $x_0 m_*$, utilise all assimilated food for maintenance and growth until they reach maturation at size m_*, from where on they invest a proportion $(\eta m/m_*)^{1-n}$ of available energy into reproduction. Such a maturation schedule implies von Bertalanffy growth trajectories for $m > m_*$ [2]. Equation (7) above follows from a metabolic model according to formula (9) of [2] in the limit of infinitely fast maturation. In this limit, a complicated but explicit expression for the Fourier transformation of $\tilde{\beta}(u)$ can be computed using symbolic algebra software, which is an important step when studying the SSSM analytically. The graph of $\tilde{\beta}(u)$ is shown in Supplementary Figure 2. In this graph, the sudden increase of biomass density above $m = m_*$ reflects the sudden slowing down of growth after maturation (as on a congested highway). The subsequent decline of density results from the thinning of ever-slower growing cohorts by predation. The ratio of offspring to maturation body mass x_0 was set to 0.02, consistent with results for invertebrates of around $1\,\mu\mathrm{gC}$ from an independent meta-analysis [15, Fig. 3]

The dynamic maturation body-mass range is chosen as $m_{*\min} = 20\,\mathrm{pgC}$, $m_{*\max} = 10\,\mathrm{kgC}$, representing a typical size range from phytoplankton to large fish in lakes.

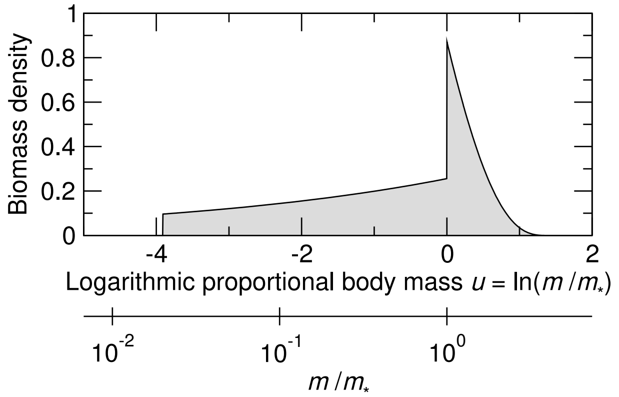

Supplementary Figure 2: Assumed distribution a population's biomass over the logarithmic body mass axis. The value $u = 0$ corresponds to the body mass at first maturation. The curve is given by Eq. (7) with $x_0 = 0.02$, $\eta = 1/4$ and $n = 3/4$.

Supplementary Table 1: Details of model definition and parameters. For an illustration of the dependencies between size-dependent quantities, see Supplementary Figure 1.

Symbol	Value	Interpretation
α	0.3	Assimilation efficiency
β	500	Preferred predator-prey mass ratio
$\tilde{\beta}(u)$	Eq. (7)	Biomass density of population on $u = \ln(m/m_*)$ axis, scaled to $\int \tilde{\beta}(u)du = 1$
γ	$10^5\,\mathrm{g}^{1-q}\mathrm{m}^{-3}\mathrm{yr}^{-1}$	Coefficient of search&attack rate
λ	$2 + q - n$	Size-spectrum exponent in oligotrophic regime
$\mu_p(m_p)$	$\int_0^\infty s\left(\ln \frac{m}{m_p}\right)[1 - f(m)]\gamma m^q \mathcal{N}(m)dm$	Predation mortality of species of size m_p
σ_r	0.5	Body size range for food-web effects
σ_s	1.5	Width of predator-prey size-ratio window
η	0.25	Maturation- over asymptotic body mass
$\phi(m)$	$\int_0^\infty m_p \mathcal{N}(m_p) s\left(\ln \frac{m}{m_p}\right) dm_p$	Food available to individuals of size m
$\tilde{B}_{\text{tot}}$	Eq. (6a)	Coefficient of ideal oligotrophic power-law species size spectrum

(continued on next page)

Supplementary Table 1 (continued)

Symbol	Value	Interpretation
$f(m)$	$\dfrac{\gamma m^q \phi(m)}{\gamma m^q \phi(m) + h m^n}$	Feeding level of species of size m
h	$85\,\mathrm{g}^{1-n}\mathrm{yr}^{-1}$	Coefficient of maximal food intake
k	$2.5\,\mathrm{g}^{1-n}\mathrm{yr}^{-1}$	Coefficient of metabolic loss rate
$\mathcal{N}(m)$	$\int_0^\infty B(m_*) W_{m_*}(m) dm_*$	Community size spectrum
m	variable	Individual body mass
m_p	variable	Body mass of prey individual
m_*	variable	Maturation body mass
$m_{*\text{min}}$	20 pg	Lower cutoff of species size spectrum
$m_{*\text{max}}$	10 kg	Upper cutoff of species size spectrum
n	0.75	Allometric exponent of respiration
$\tilde{\mathcal{N}}$	Eq. (6b)	Coefficient of ideal oligotrophic power-law community size spectrum
q	0.9	Allometric exponent of search/attack rate
ρ	$10\,\mathrm{g}^{1-n}\mathrm{yr}^{-1}$	Strength of food-web effects
$s(x)$	$\exp\left[-(x - \ln \beta)^2/(2\sigma_s^2)\right]$	Predator-prey mass-ratio window
$W_{m_*}(m)$	$m^{-2}\tilde{\beta}(\ln(m/m_*))$	A population's density of individuals on m-axis, scaled to $\int m W_{m_*}(m) dm = 1$
x_0	0.02	Body mass of offspring relative to m_*

Supplementary Note 3: Model Simulations

For numerical simulations of size-spectrum dynamics, the $\log_{10}(m_*)$ and $\log_{10}(m)$-axes were discretized to $N = 512$ lattice points separated by 0.05 starting from $m_* = m_{*\text{min}}$. Evaluation of $\Lambda(m_*)$, given by Eq. (1) and Supplementary Table 1, requires computation of several nested integrals over m_* and m in each simulation time step (Supplementary Figure 1). We approximated these integrals by midpoint Riemann sums over the simulation lattice, and made use of the simplifying allometric scaling assumptions for life-history traits in Supplementary Note 1 to convert these sums into discrete convolution operations, which were evaluated using fast Fourier transforms. This reduced the computational cost of the model from $\mathcal{O}(N^2)$ to $\mathcal{O}(N \log N)$ per simulation time step. The resulting system of ODEs was simulated using the solver CVODE from the SUNDIALS package [16], which automatically adjusted approximation order and step size to achieve our prescribed accuracy of 10^{-4} per step for $\ln B(m_*)$. In addition, upper limits

on step size, scaling as the square-root of time since the start of simulations, were imposed to avoid numerical instability (CAT, Sec. 8.2).

The initial condition for each simulation was taken to be x times the ideal oligotrophic power-law size spectrum $\tilde{B}_{\mathrm{tot}} m_*^{1-\lambda}$. Because for large x the size spectra can exhibit periodic or irregular dynamics, simulations were first run with a burn-in of 20 years, before 100 snapshots taken in 1-year intervals were evaluated to compute medians and quantile ranges of the characteristics, B_0, S, A, and D in Fig. 3.

Supplementary Note 4: Empirical size spectra and fitted modulated lines

In the following, we show the graphs of all 25 empirical size spectra included in this study (open circles), together with the linear-plus-sinusoidal fits (red lines) described in the main text. Since only data up to $0.1\mathrm{gC}$ body mass were used for the model fits (Materials Methods), the red lines are restricted to this range. For easy comparison, all graph have identical horizontal axes and identically scaled vertical axes. The spectra are shown in the order in which they were listed in Materials and Methods: approximately sorted by increasing nutrient concentration, while keeping related spectra together.

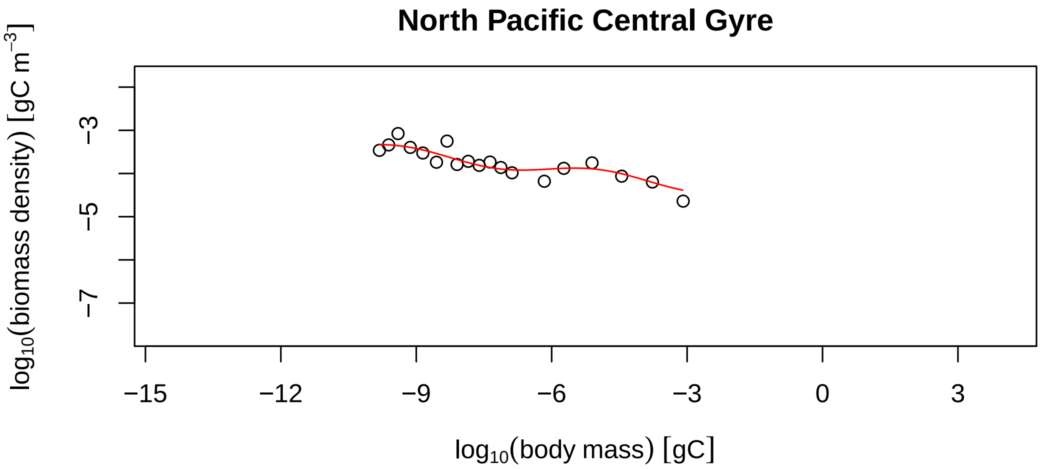

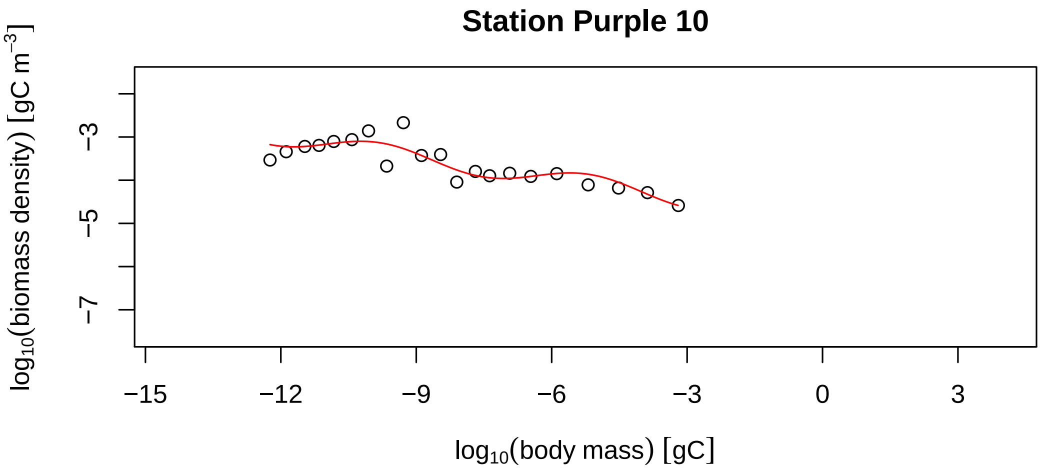
Station Purple 10
log10(biomass density) [gC m−3]
−3
−5
−7
−15
−12
−9
−6
−3
0
3
log10(body mass) [gC]

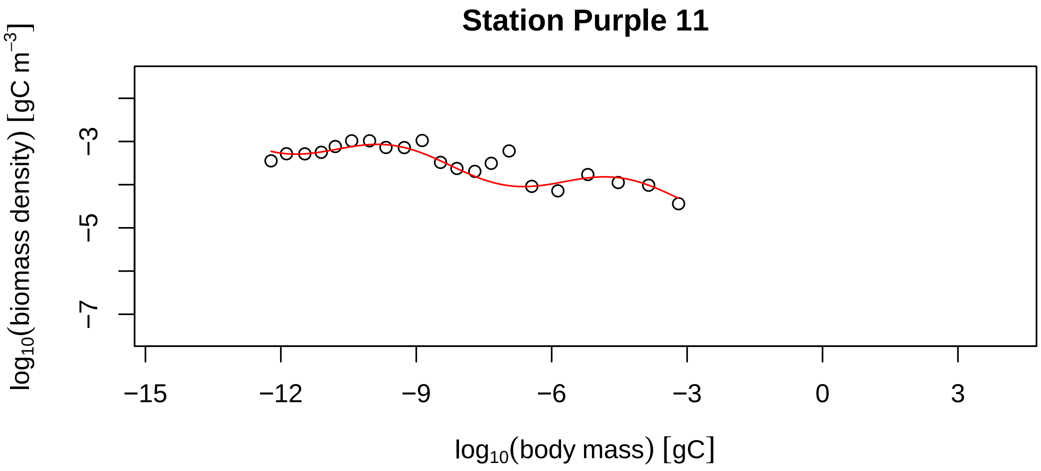
Station Purple 11
log10(biomass density) [gC m−3]
−3
−5
−7
−15
−12
−9
−6
−3
0
3
log10(body mass) [gC]

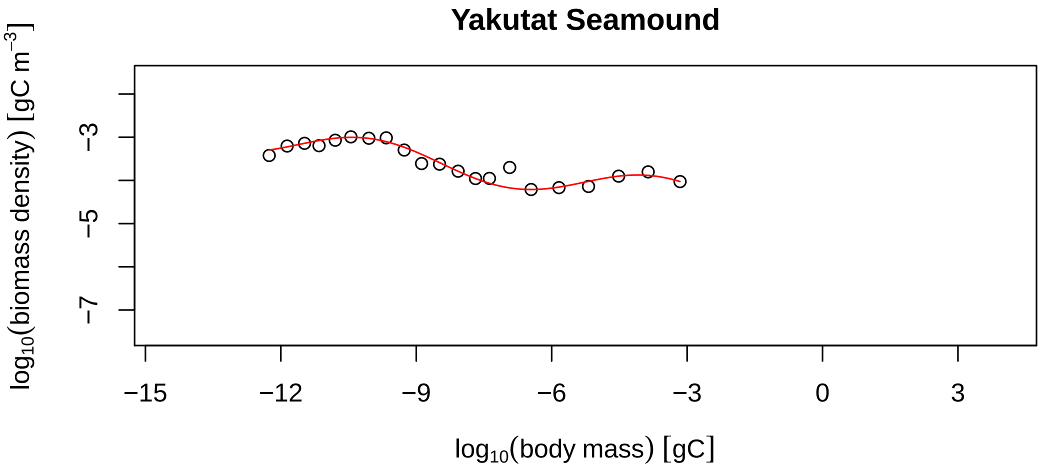
Yakutat Seamound
log10(biomass density) [gC m−3]
−3
−5
−7
−15
−12
−9
−6
−3
0
3
log10(body mass) [gC]

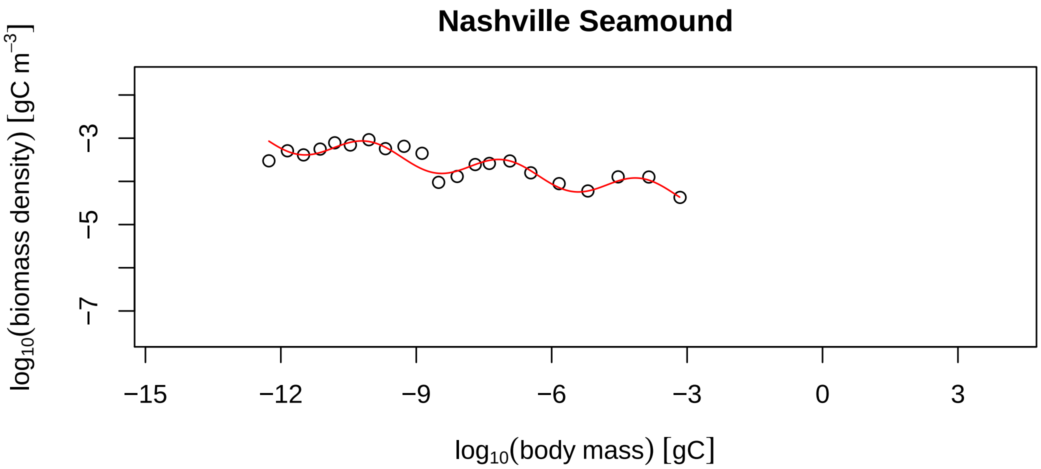

Nashville Seamound
log10(biomass density) [gC m−3]
−3
−5
−7
−15
−12
−9
−6
−3
0
3
log10(body mass) [gC]

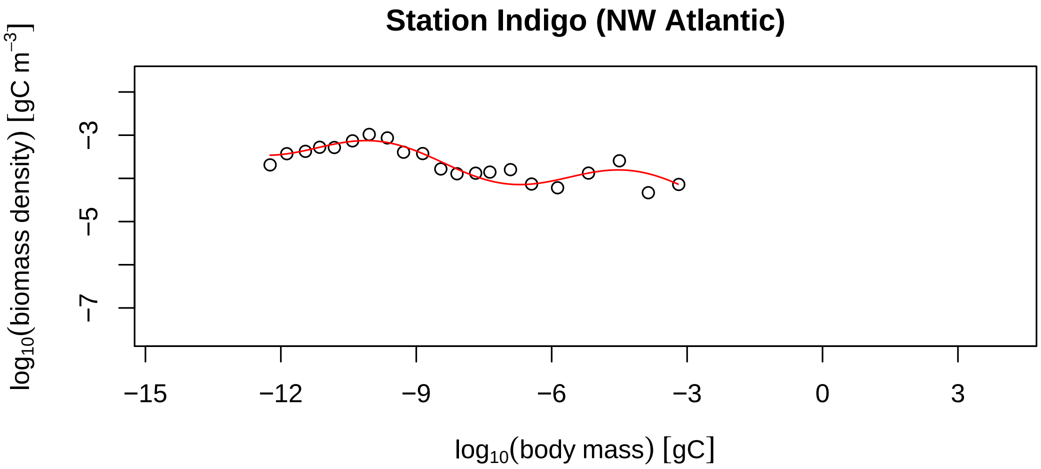

Station Indigo (NW Atlantic)
log10(biomass density) [gC m−3]
−3
−5
−7
−15
−12
−9
−6
−3
0
3
log10(body mass) [gC]

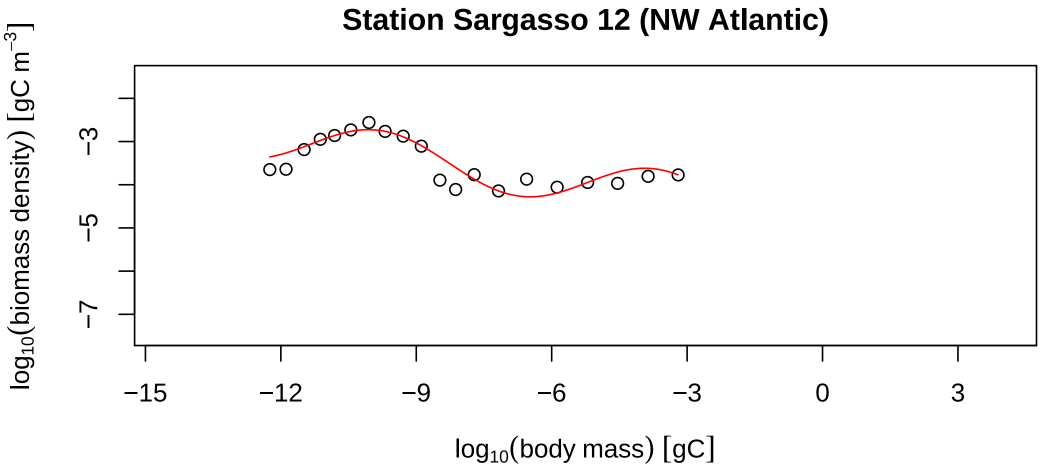

Station Sargasso 12 (NW Atlantic)
log10(biomass density) [gC m−3]
−3
−5
−7
−15
−12
−9
−6
−3
0
3
log10(body mass) [gC]

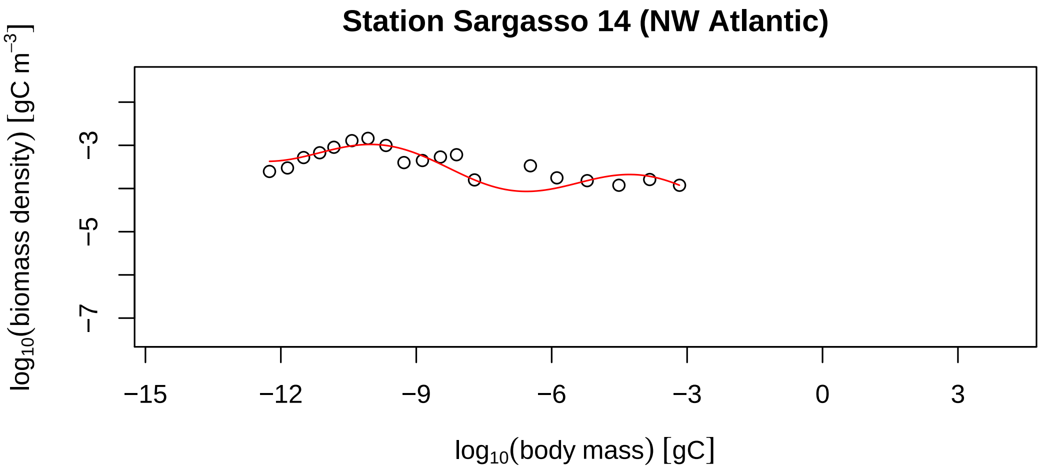
Station Sargasso 14 (NW Atlantic)
log10(biomass density) [gC m−3]
−3
−5
−7
−15
−12
−9
−6
−3
0
3
log10(body mass) [gC]

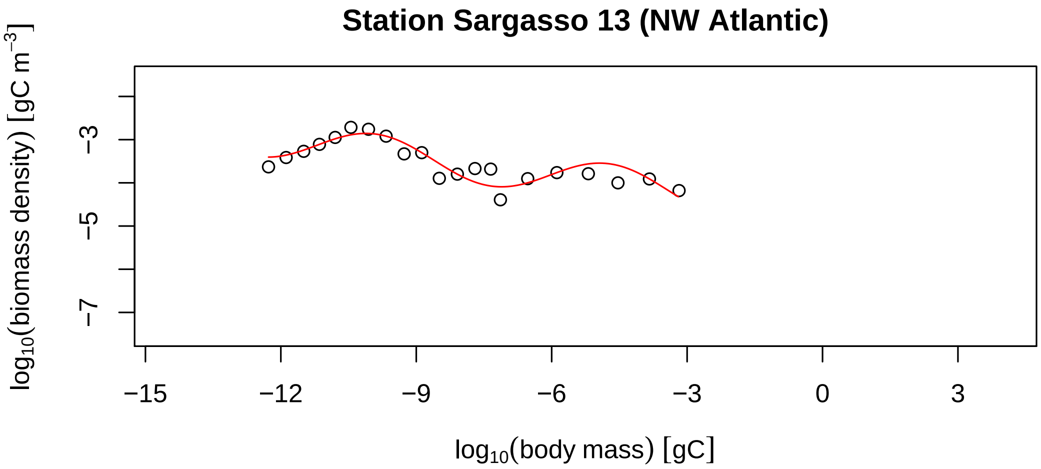
Station Sargasso 13 (NW Atlantic)
log10(biomass density) [gC m−3]
−3
−5
−7
−15
−12
−9
−6
−3
0
3
log10(body mass) [gC]

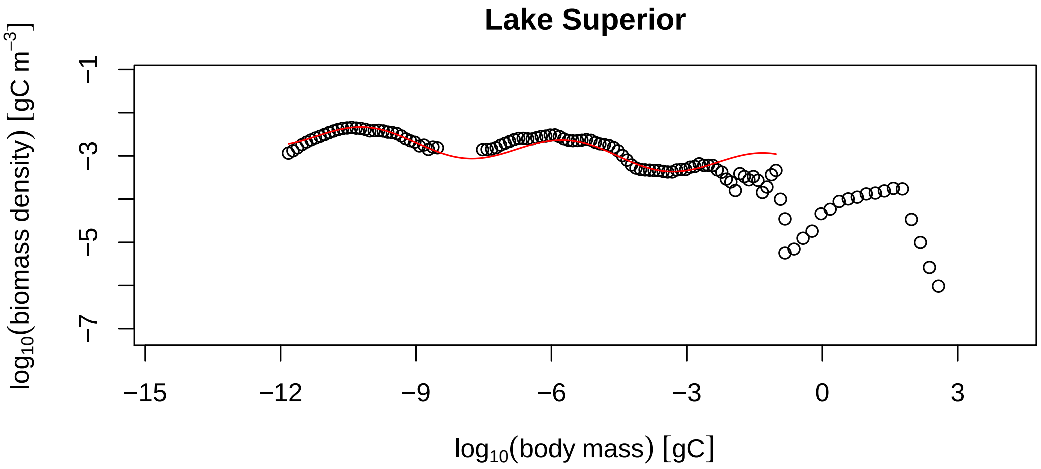
Lake Superior
log10(biomass density) [gC m−3]
−1
−3
−5
−7
−15
−12
−9
−6
−3
0
3
log10(body mass) [gC]

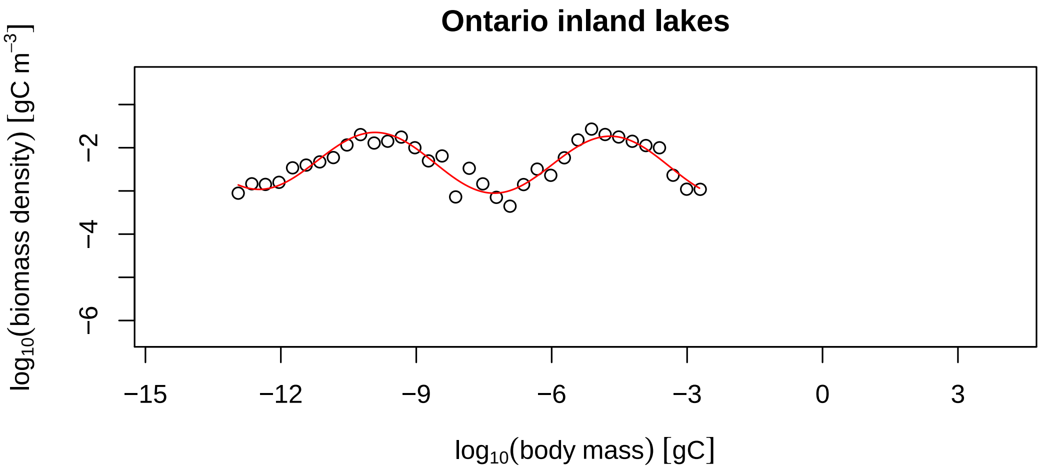

Ontario inland lakes
log10(biomass density) [gC m−3]
−2
−4
−6
−15
−12
−9
−6
−3
0
3
log10(body mass) [gC]

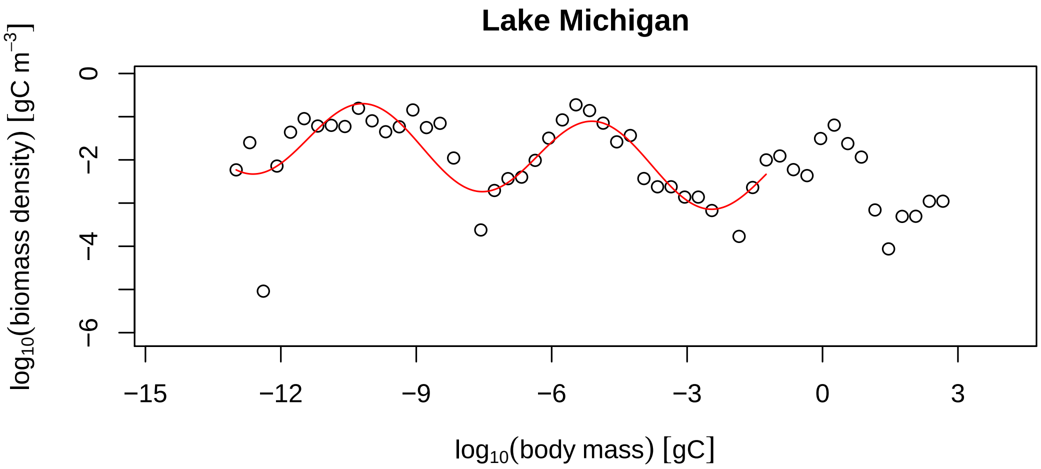

Lake Michigan
log10(biomass density) [gC m−3]
0
−2
−4
−6
−15
−12
−9
−6
−3
0
3
log10(body mass) [gC]

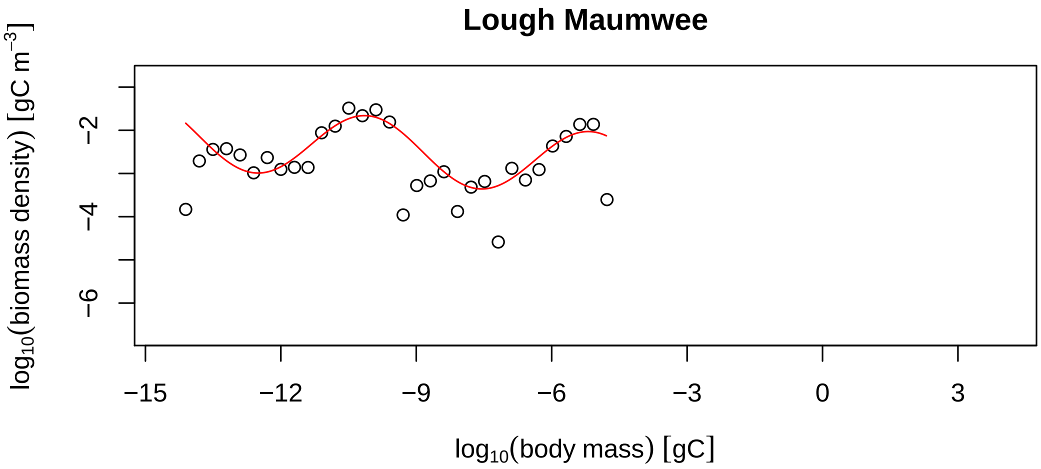

Lough Maumwee
log10(biomass density) [gC m−3]
−2
−4
−6
−15
−12
−9
−6
−3
0
3
log10(body mass) [gC]

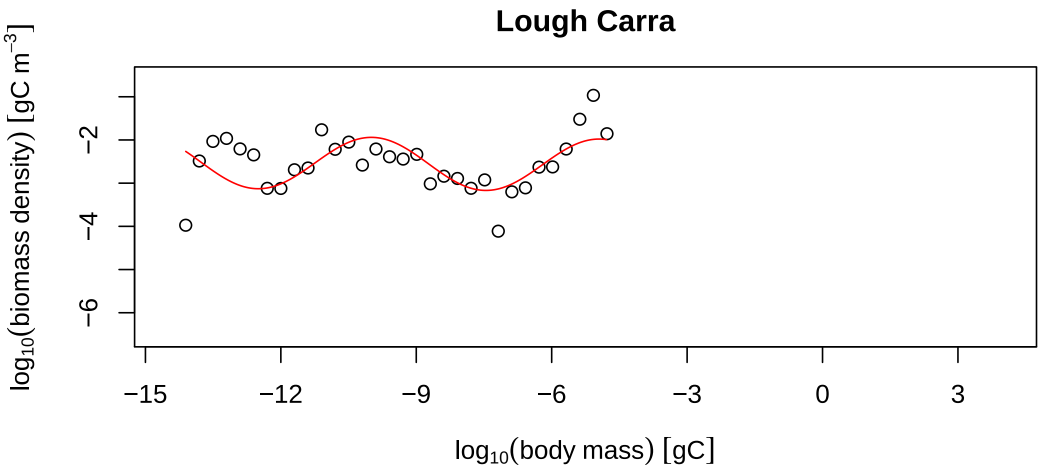
Lough Carra
log10(biomass density) [gC m−3]
−6
−4
−2
−15
−12
−9
−6
−3
0
3
log10(body mass) [gC]

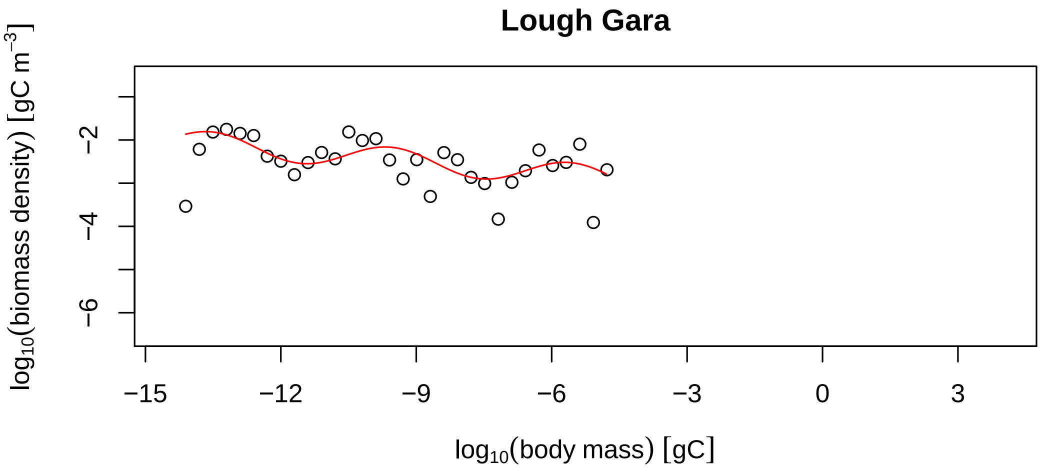
Lough Gara
log10(biomass density) [gC m−3]
−6
−4
−2
−15
−12
−9
−6
−3
0
3
log10(body mass) [gC]

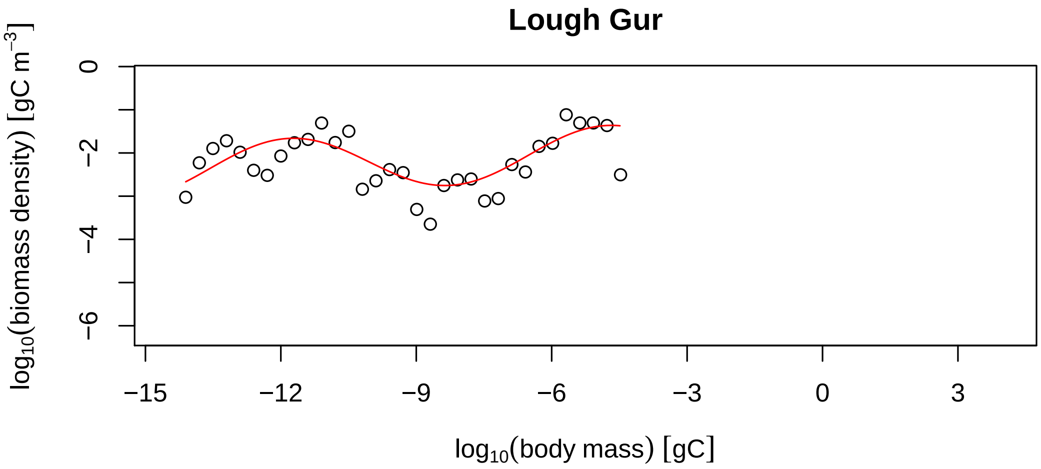
Lough Gur
log10(biomass density) [gC m−3]
−6
−4
−2
0
−15
−12
−9
−6
−3
0
3
log10(body mass) [gC]

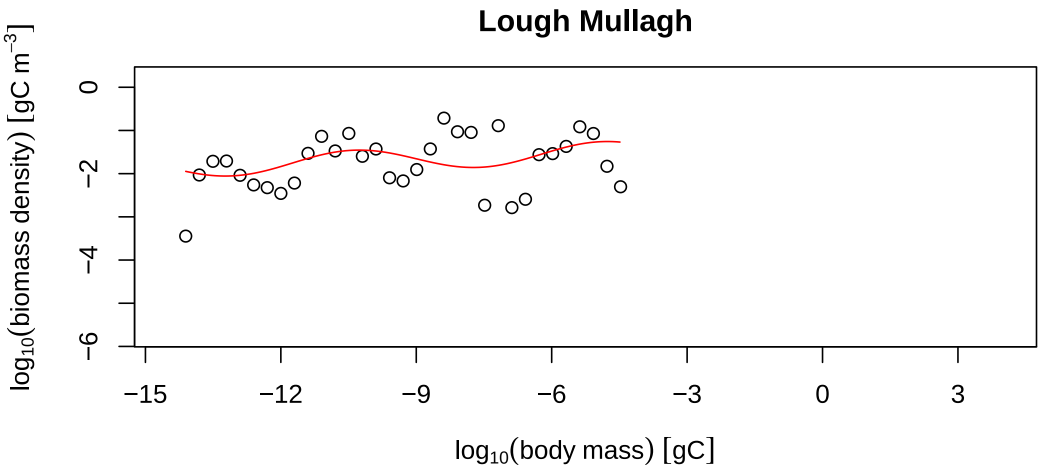
Lough Mullagh
log10(biomass density) [gC m−3]
0
−2
−4
−6
−15
−12
−9
−6
−3
0
3
log10(body mass) [gC]

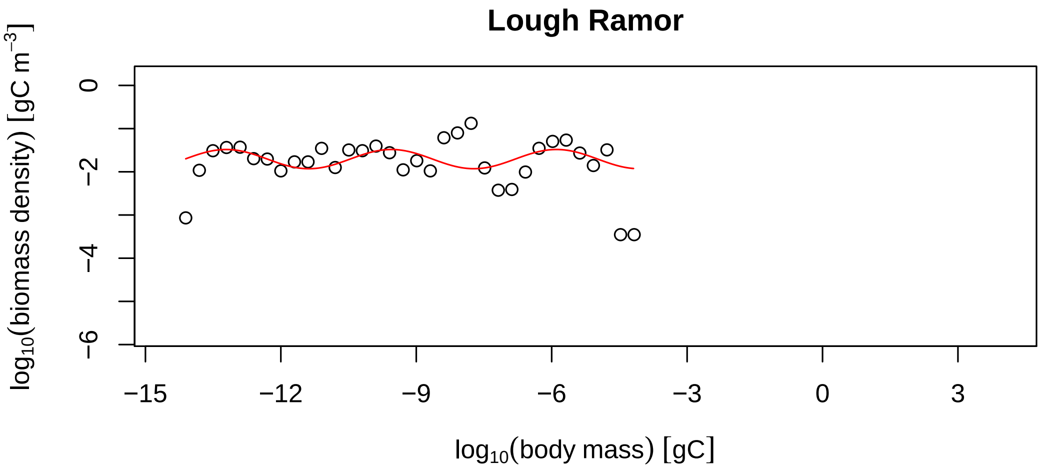
Lough Ramor
log10(biomass density) [gC m−3]
0
−2
−4
−6
−15
−12
−9
−6
−3
0
3
log10(body mass) [gC]

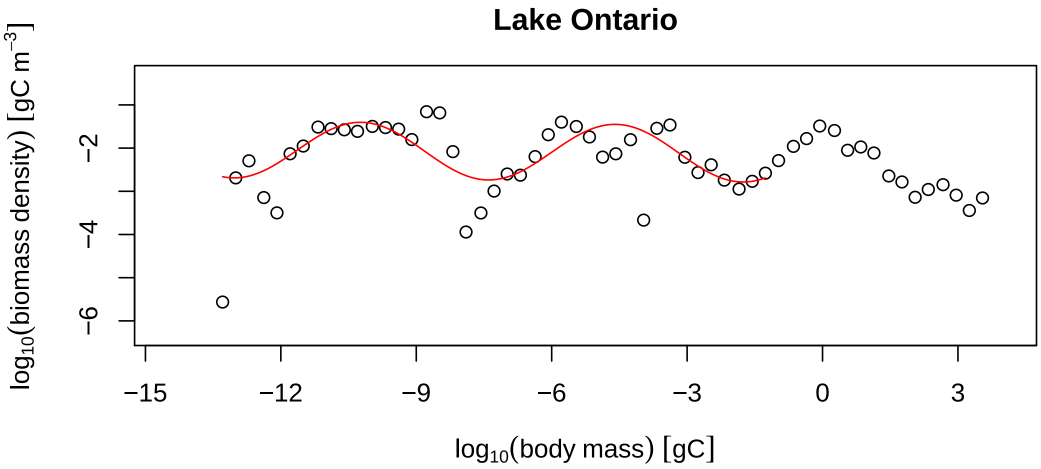
Lake Ontario
log10(biomass density) [gC m−3]
−2
−4
−6
−15
−12
−9
−6
−3
0
3
log10(body mass) [gC]

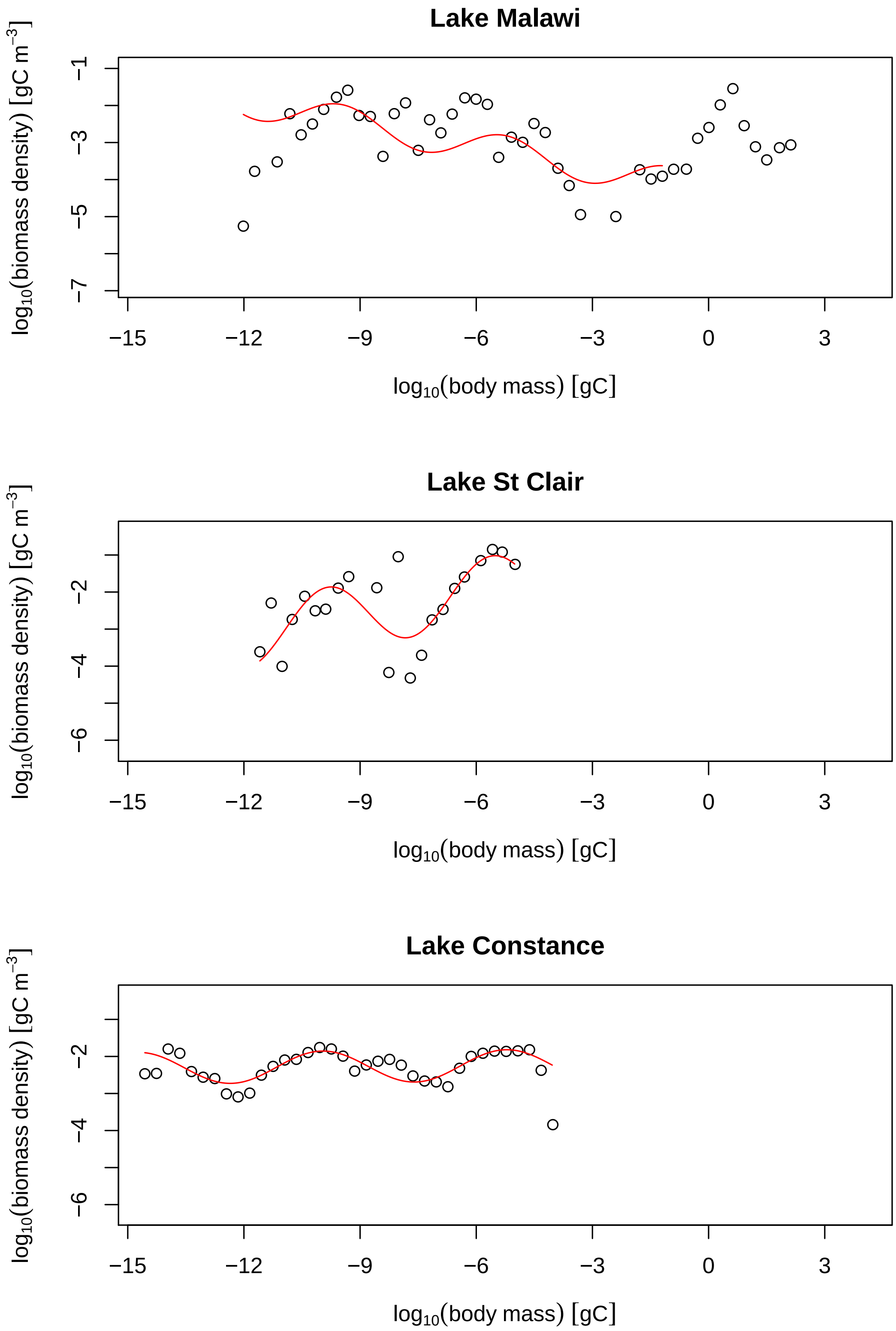
Lake Malawi
log10(biomass density) [gC m−3]
log10(body mass) [gC]
Lake St Clair
log10(biomass density) [gC m−3]
log10(body mass) [gC]
Lake Constance
log10(biomass density) [gC m−3]
log10(body mass) [gC]

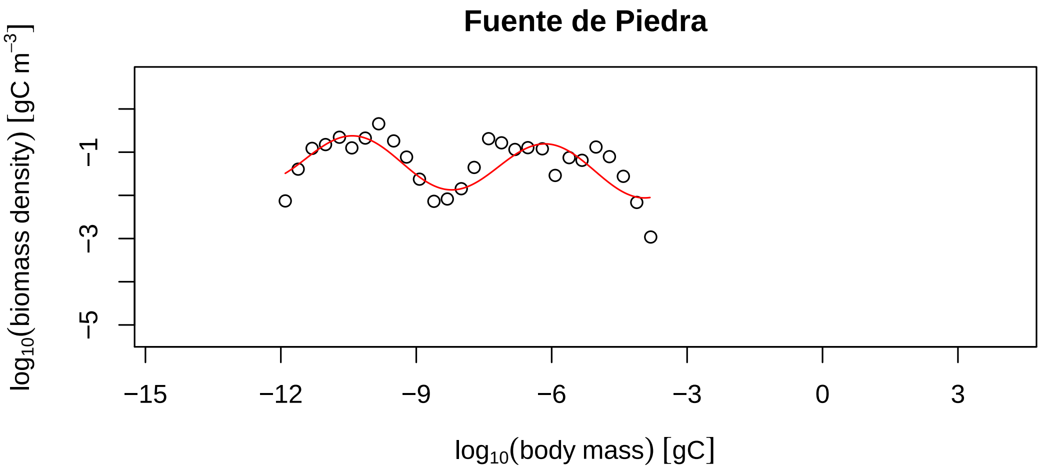

Fuente de Piedra
log10(biomass density) [gC m−3]
−1
−3
−5
−15
−12
−9
−6
−3
0
3
log10(body mass) [gC]

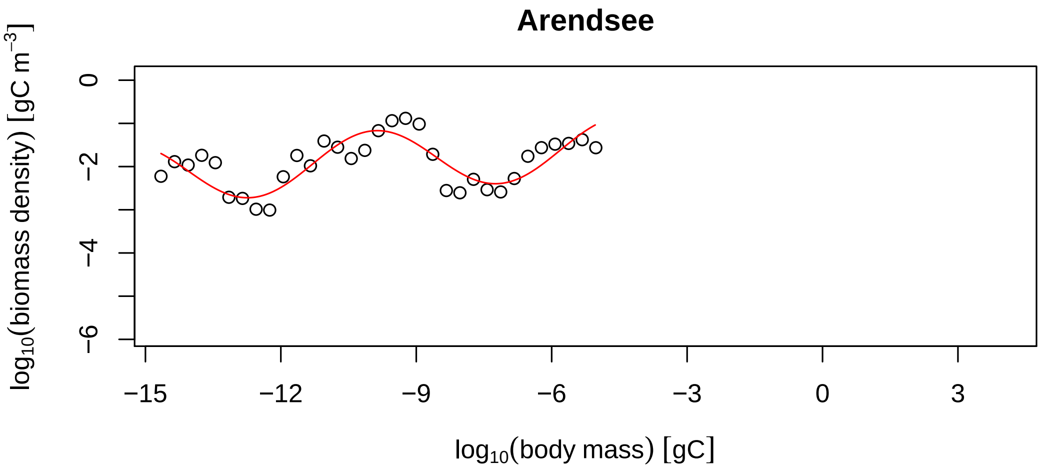

Arendsee
log10(biomass density) [gC m−3]
0
−2
−4
−6
−15
−12
−9
−6
−3
0
3
log10(body mass) [gC]

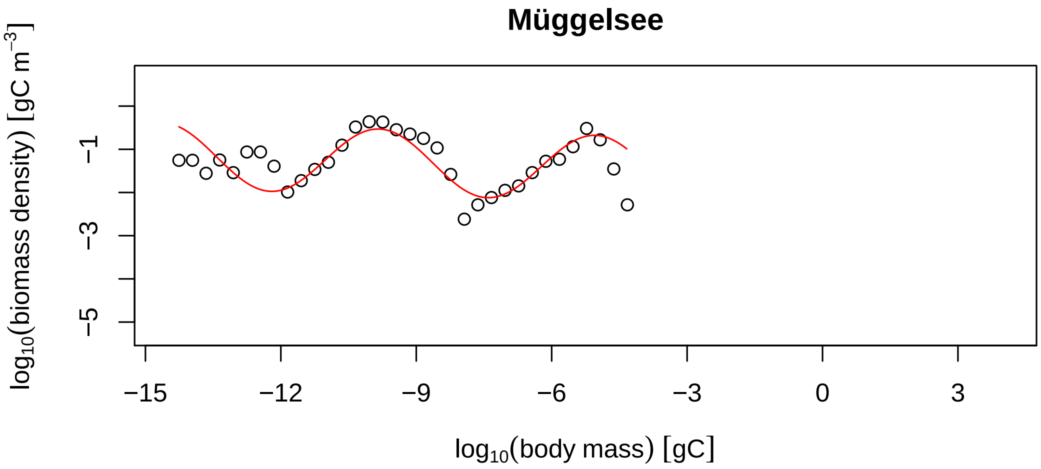

Müggelsee
log10(biomass density) [gC m−3]
−1
−3
−5
−15
−12
−9
−6
−3
0
3
log10(body mass) [gC]

Characteristic		Scale	c_0	c_1	c_1/c_0	p	$\mathrm{corr}(c_0, c_1)$
Intercept	B_0	$\log_{10}(\mathrm{gC\,m^{-3}})$	$-4.25(11)$	$1.53(14)$		9.5×10^{-11}	-0.42
Slope	S	dimensionless	$-0.1197(71)$	$0.0789(86)$	-0.66	3.5×10^{-09}	-0.4
Amplitude	A	$\log_{10}(\mathrm{biomass})$	$0.363(36)$	$0.095(32)$	0.26	0.0068	-0.6
Separation	D	$\log_{10}(\mathrm{body\ mass})$	$5.30(19)$	$-0.34(16)$	-0.064	0.042	-0.81

Supplementary Table 2: For each characteristic $C = B_0$, S, A, D, the table gives details of the weighted linear regression $C = c_0 + c_1 \log_{10}(\mathrm{TP}/\mu\mathrm{g\,L^{-1}})$. Figures in parentheses represent standard errors of regression coefficients, p relates to the null-hypothesis $c_1 = 0$ (two-tailed).

Supplementary Note 5: Regressions of empirical size-spectrum characteristics *vs* nutrient enrichment

In Supplementary Table 2, we provide details of weighted linear regressions of the four empirical size-spectrum characteristics B_0, S, A, and D against $\log_{10}$ TP. Weights were taken as the inverse squared standard errors of data points. The weighted regressions were computed using the lm function of the R programming language [17, version 3.4.2]. The ratio c_1/c_0 is tabulated as a measure of effect strength, excluding the case of B_0 where this value depends on the units of measurement chosen. This shows that the effect of nutrient enrichment on the separation between domes D is weak compared to the effects on slope S and dome amplitude A.

Supplementary Note 6: The linear SSSM and its responses to size-specific pressures

In Supplementary Note 7 below we present an analysis showing that domes in the SSSM are generated by an amplifying top-down cascade resulting from nutrient enrichment. The analysis is carried out using the linearized version of the model described above, the linear SSSM. In the present section the linear SSSM and the linear response theory derived for it in CAT are briefly recalled.

6.1 Formulation of the linear SSSM

Two variants of the linear SSSM have been analysed in CAT. They were there referred to as the "oligotrophic" and the "eutrophic" regime. Here, we consider the oligotrophic regime, defined in Supplementary Note 1 above. In this regime, dynamics are linearized around an idealised equilibrium model state $B(m_*) = B_0(m_*)$ of the form

$$B_0(m_*) = \tilde{B}_{\mathrm{tot}} m_*^{1-\lambda} \quad \text{(for all } m_* > 0), \tag{8}$$

with $\tilde{B}_{\mathrm{tot}}$ given by Eq. (6).

The linear SSSM is best formulated using instead of m_* the logarithmic maturation body mass variable $u = \ln(m_*/M)$, with M denoting some reference body mass, e.g. $1\,\mathrm{gC}$. The distribution of a community's biomass over the u-axis is given by $m_* B(m_*) = M\mathrm{e}^u B(M\mathrm{e}^u)$. We follow CAT and denote by $b(u)$ deviations of this distribution from that given by the base state, that is

$$b(u) = M\mathrm{e}^u B(M\mathrm{e}^u) - M\mathrm{e}^u B_0(M\mathrm{e}^u). \tag{9}$$

The linear SSSM is an integro-differential equation for the dynamics of $b(u)$. Absent any external pressures, they are given by

$$\frac{\partial b(u)}{\partial t} = (M\mathrm{e}^u)^{n-1} \int_{-\infty}^{\infty} \tilde{K}(u-v) b(v) dv. \tag{10}$$

In this equation, the interaction kernel $\tilde{K}(w)$ describes for any $w = u - v$ the strength of the effect of populations of species of size $M\mathrm{e}^v$ on a focal species of size $M\mathrm{e}^u$. The parameter w is thus a logarithmic size ratio $w = \ln[(M\mathrm{e}^u)/(M\mathrm{e}^v)]$. The interaction kernel $\tilde{K}(w)$ incorporates all the "ecology" of the model. In the SSSM, it contains contributions due to predation mortality and density-dependent growth, and a term describing damping due to food-web effects, corresponding to that in Eq. (3) above. An explicit formula will be given in Supplementary Note 7 below. The factor $(M\mathrm{e}^u)^{n-1}$ in Eq. (10) models the size dependence of the time scale of demographic processes: populations of smaller species tend to change faster than those of larger species (note that $n < 1$, Supplementary Table 1).

6.2 Fourier transforms

Below we shall make heavy use of Fourier transforms of continuous functions. Here we specify the particular form in which we define Fourier transforms, because conventions for this vary in the literature. For any function $f(x)$ defined over the real numbers, we define its Fourier transform $\hat{f}(\xi)$ such that, at least formally,

$$\hat{f}(\xi) = \int_{-\infty}^{\infty} \mathrm{e}^{-\mathrm{i}\xi x} f(x)\, dx, \quad f(x) = \int_{-\infty}^{\infty} \frac{\mathrm{e}^{\mathrm{i}\xi x}}{2\pi} \hat{f}(\xi)\, d\xi, \tag{11}$$

where i is the imaginary unit ($\mathrm{i}^2 = -1$) and ξ is a real- or complex-valued variable. For a gentle but mathematically rigorous discussion of Fourier transforms and related topics, see e.g. [18].

6.3 Linear response theory for the SSSM

How will the species size spectrum respond when species *of a specific size* are subjected to a persistent, constant pressure, such as continuous removal, stocking, or feeding, i.e., a press

perturbation? What is the new equilibrium reached after all ecological interactions up and down the size axis have played out? A general analysis of equations of the form (10) suggests that the following recipe answers this question (CAT, Sec. 8.4.3):

1. Compute from $\tilde{K}(w)$ its Fourier transform $\hat{K}(\xi)$.

2. Obtain the analytic continuation of $\hat{K}(\xi)$ into the complex plane (if an explicit expression for $\hat{K}(\xi)$ is available, this usually amounts to simply permitting ξ to attain any complex value, up to a few values where $\hat{K}(\xi)$ might be singular).

3. Find the first few zeros of $\hat{K}(\xi)$ in the complex plane (the values of ξ where $\hat{K}(\xi) = 0$) in order of increasing $|\xi|$, and evaluate the derivatives $\hat{K}'(\xi) = d\hat{K}(\xi)/d\xi$ at these zeros.

4. Draw a graph of the complex plane, indicating in it each zero of $\hat{K}(\xi)$ by an arrow pointing from the zero ξ into the direction $(\,\mathrm{Re}[\hat{K}'(\xi)],\,\mathrm{Im}[\hat{K}'(\xi)]\,)$. Then draw the line $\mathrm{Im}(\xi) = -(2-\lambda) = \lambda - 2$, with λ defined as in Supplementary Table 1. An example of such a graph is given in Supplementary Figure 3. For the representation of size spectra used here, the value $2-\lambda$ corresponds to the slope (actually: power-law exponent) of the ideal power-law size spectrum in the oligotrophic regime.

5. From this graph, read off the responses of the species size spectrum to a press perturbations on a give size class as follows. The zeros come in pairs $\pm\,\mathrm{Re}(\xi) + \mathrm{i}\,\mathrm{Im}(\xi)$, unless $\mathrm{Re}(\xi) = 0$. Each of these pairs or imaginary singletons corresponds to one type of response. If the attached arrow points upwards, it is a top-down response, where species smaller that those perturbed are being affected. If the attached arrow points downward, it is a bottom-up response.

   Purely imaginary zeros correspond to un-modulated size-spectrum responses. In all other cases the response is modulated, i.e. a "cascade". The wavelength of the modulation along the u axis is $|2\pi/\,\mathrm{Re}(\xi)|$. That is, neighbouring maxima (e.g. the domes) occur for species with maturation body sizes differing by an approximate factor $\exp(|2\pi/\,\mathrm{Re}(\xi)|)$.

   If the attached arrow points towards the line $\mathrm{Im}(\xi) = \lambda - 2$, the response is attenuating, i.e. the proportional change in $B(m_*)$ tends to become smaller the more m_* is different from the size of the perturbed species. Conversely, if the attached arrow points away from the line $\mathrm{Im}(\xi) = \lambda - 2$, the response is amplifying, i.e. the proportional change in $B(m_*)$ tends to become larger the more m_* is different from the size of the perturbed species. Quantitatively, the amplitude of the proportional change in $B(m_*)$ for species that are by a factor Z larger or small than those perturbed is by a factor $Z^{|\,\mathrm{Im}(\xi)-\lambda+2|}$ larger or smaller than that of the perturbed species.

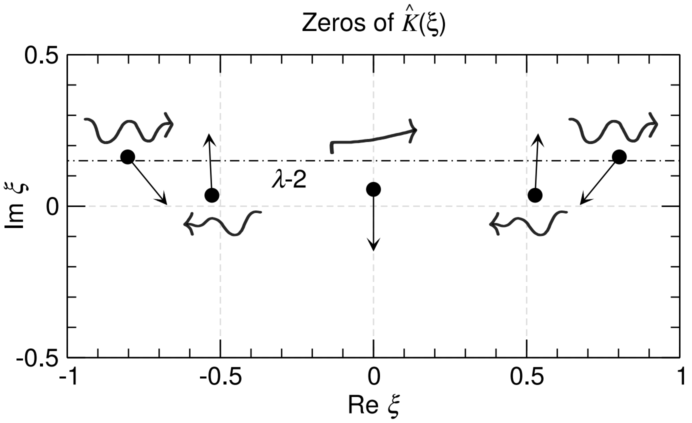

Supplementary Figure 3: Points in the complex plane corresponding to the main responses of the SSSM to press perturbations. Corresponding size-spectrum responses are illustrated. From the centre outwards, these are the conventional bottom-up effect, the conventional top-down cascade, and a bottom-up cascade. Parameters as in Supplementary Table 1 with $x = 1$ (which implies $y = 1$ below).

In Supplementary Figure 3, which corresponds the parameter set used in the main text (Supplementary Table 1) with $x = 1$, one sees, in order of increasing distance from the origin of the complex plane, one imaginary singleton and two complex pairs. These are the three kinds of responses of size spectra to pressures mentioned in the main text:

1. The singleton at $\xi = 0 + 0.055\,\mathrm{i}$ has a downward-pointing arrow attached. It therefore corresponds to an un-modulated bottom-up effect. Because it points away from the dashed-dotted line, the effect is amplifying. This is the conventional bottom-up effect with trophic amplification. [The ecological reason for trophic amplification is that more abundant prey can not only sustain more predators, the prey is also easier to find; see 6, Section 21.2.2.]

2. The pair of zeros at $\xi = \pm 0.528 + 0.034\,\mathrm{i}$ has upward-pointing arrows attached. It therefore corresponds to a modulated top-down effect. Because the arrows point towards the dashed-dotted line, the effect is attenuating. This is the conventional trophic cascade, here with effect strengths declining towards lower trophic levels, as observed for marine pelagic systems [19].

3. The pair at $\xi = \pm 0.803 + 0.163\,\mathrm{i}$ has downward-pointing arrows attached. It therefore corresponds to a modulated bottom-up effect, i.e. a bottom-up cascade as predicted by the classical theory [20, 21, 22]. Because these zeros are (for the chosen parameters) located very close to the line $\mathrm{Im}\,\xi = \lambda - 2 = 0.15$ (cf. Supplementary Table 1), the cascade is

effectively neither amplifying not attenuating. However, with increasing nutrient levels it gets "drowned out" by trophic amplification (see below).

Zeros even farther away from the origin appear to always correspond to attenuating responses (e.g. Supplementary Figure 6). They are less important ecologically, because with increasing $|\operatorname{Re}\xi|$ the corresponding wavelength on the u-axis becomes shorter and with increasing $|\operatorname{Im}\xi|$ attenuation becomes stronger, which makes them relevant only for highly size-specific pressures and only for species of size very similar to those where pressure is applied.

6.4 Demonstration of the recipe on a simple example

The method described above has been verified numerically in CAT. In this section, we demonstrate it for a highly simplified, exactly solvable problem as a transparent illustration of how the method works. We will first introduce the example problem and its exact solution, and then compare this solution with the predictions made in Sec. 6.3 above.

Consider the simple drift-diffusion-growth equation,

$$\frac{df(x,t)}{dt} = rf(x,t) - v\frac{df(x,t)}{dx} + D\frac{d^2 f(x,y)}{dx^2}, \tag{12}$$

which specifies how the distribution of some quantify $f(x,t)$ over the x axis how it changes through time t. The three terms on the right-hand-side describe self-reproduction at a rate r, drift along the x axis with velocity v and diffusion at a rate $D > 0$. This equation has, amongst others, solutions of the form of drifting Gaussians

$$f(x,t) = f_{\mathrm{G}}(x,t;f_0,\sigma_0) \stackrel{\mathrm{def}}{=} \frac{f_0 \mathrm{e}^{rt}}{\sqrt{2\pi(2Dt+\sigma_0^2)}} \exp\left[-\frac{(x-vt)^2}{4Dt+2\sigma_0^2}\right], \tag{13}$$

with free parameters f_0 and σ_0. Now, consider a modification of Eq. (12) where for $t \geq 0$ a Gaussian inhomogeneity of width σ centred at $x = 0$, representing a press perturbation, is added to its right-hand side:

$$\frac{df(x,t)}{dt} = rf(x,t) - v\frac{df(x,t)}{dx} + D\frac{d^2 f(x,y)}{dx^2} + \frac{\mathrm{e}^{-x^2/(2\sigma^2)}}{\sqrt{2\pi}\sigma} \quad (\text{for } t \geq 0). \tag{14}$$

If one assumes $f(x,t) \equiv 0$ for $t < 0$, this equation has for $t \geq 0$ a unique solution that can be represented by an integral over drifting Gaussians that originated from the inhomogeneity during the times τ between zero and t:

$$f(x,t) = f_{\mathrm{I}}(x,t) \stackrel{\mathrm{def}}{=} \int_0^t f_{\mathrm{G}}(x,t-\tau;1,\sigma)d\tau. \tag{15}$$

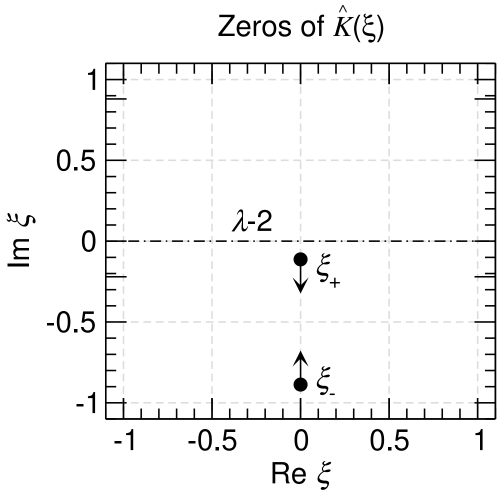

Supplementary Figure 4: The two points in the complex plane corresponding to zeros of the interaction kernel for the simple model of Sec. 6.4. Parameters $v = 1$, $D = 1$, $r = 0.1$. The two zeros are given by Eq. (21); $\lambda = 2$.

In the limit $\sigma \to 0$, this integral evaluates to

$$f_{\mathrm{I}}(x,t) = \frac{\mathrm{e}^{(xv-|x|\sqrt{v^2-4Dr})/(2D)}}{2\sqrt{v^2-4Dr}}\,\mathrm{erfc}\left(\frac{|x|-t\sqrt{v^2-4Dr}}{2\sqrt{Dt}}\right) + \frac{\mathrm{e}^{(xv+|x|\sqrt{v^2-4Dr})/(2D)}}{2\sqrt{v^2-4Dr}}\,\mathrm{erfc}\left(\frac{|x|+t\sqrt{v^2-4Dr}}{2\sqrt{Dt}}\right), \quad (16)$$

with erfc denoting the complementary error function [23]. This solution can be verified directly by inserting it into Eq. (14) and taking the limit $\sigma \to +\infty$. In the following, we concentrate on the case $4Dr < v^2$, where the arguments of the error functions are real-valued. For real arguments y, the function $\mathrm{erfc}(y)$ transitions smoothly from 2 to 0 as y passes from negative to positive values. The first term in Eq. (16) therefore describes two fronts that propagate at a speed $\pm\sqrt{v^2-4Dr}$ away from zero. The first occurrence of erfc in Eq. (16) approaches 2 after sufficiently long waiting times t for any fixed value of x. The second occurrence converges to zero as t increases. The solution therefore reaches the steady state

$$\lim_{t\to+\infty} f_{\mathrm{I}}(x,t) = \frac{\mathrm{e}^{(xv-|x|\sqrt{v^2-4Dr})/(2D)}}{\sqrt{v^2-4Dr}}. \quad (17)$$

We now show that this result is consistent with the recipe of Sec. 6.3 when adapting it to Eq. (12). The equation can be rewritten as

$$\frac{df(x,t)}{dt} = \int_{-\infty}^{+\infty} K(x-y) f(y,t) dy, \tag{18}$$

with an interaction kernel

$$K(x) = r\delta(x) - v\delta'(x) + D\delta''(x), \tag{19}$$

where $\delta(x)$ represents the Dirac delta function. Complications due to deviations from Sheldon's hypothesis [24] ($\lambda \neq 2$) are not relevant here, we therefore set $\lambda = 2$ when applying the recipe.

Step 1 of the recipe is to compute the Fourier transform of $K(x)$. According to Eq. (11) this is

$$\hat{K}(\xi) = r - \mathrm{i}v\xi - D\xi^2. \tag{20}$$

Because $\hat{K}(\xi)$ is an entire function in ξ, its analytic continuation into the complex plane, required by Step 2, is formally identical.

Step 3 requires computation of the zeros of $\hat{K}(\xi)$. As for the direct solution of Eq. (14) above, we concentrate on the case $4Dr < v^2$. Further, we consider only the case $v > 0$; the analysis for $v < 0$ is analogous. Under these conditions, $\hat{K}(\xi)$ has two purely imaginary zeros given by

$$\xi_+ = \frac{-v + \sqrt{v^2 - 4Dr}}{2D}\,\mathrm{i} \qquad \text{and} \qquad \xi_- = \frac{-v - \sqrt{v^2 - 4Dr}}{2D}\,\mathrm{i}, \tag{21}$$

with $|\xi_+| < |\xi_-|$. If r has a value close to zero one can approximate $\xi_+ \approx -\frac{r}{v}\mathrm{i}$ and $\xi_- \approx (-\frac{v}{D} + \frac{r}{v})\mathrm{i}$ to first order in r. That is, ξ_+ then tends to be considerably closer to zero then ξ_- and therefore more important for responses to press perturbations according to the reasoning of Sec. 6.3. At the zeros, the first derivative $\hat{K}'(\xi) = -\mathrm{i}v - 2D\xi$ evaluates to

$$\hat{K}'(\xi_+) = -\sqrt{v^2 - 4Dr}\, i \qquad \text{and} \qquad \hat{K}'(\xi_-) = \sqrt{v^2 - 4Dr}\, i. \tag{22}$$

Figure 4 shows the graph drawn following the instructions of Step 4 for parameters $v = 1$, $D = 1$, $r = 0.1$. Interpreting this graph according to Step 5 *mutatis mutandis*, the arrow for ξ_+ predicts a response that propagates towards larger x (because the arrow points downwards). It is amplifying because it points away from the semi-dashed line (here at $\mathrm{Im}\,\xi = 0$). Correspondingly, the arrow for ξ_- predicts an attenuating response that propagates towards smaller x. Because ξ_- is considerably further away from zero than ξ_+, the decay of the attenuating response is comparatively fast. The corresponding perturbation response therefore does not affect overall system behaviour much.

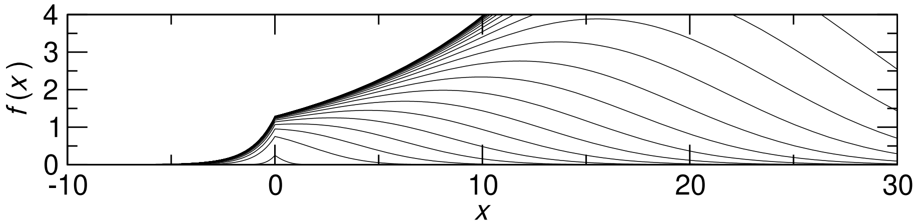

Supplementary Figure 5: Exact solution of Eq. (14) for $\sigma \to 0$, given by Eq. (16), at subsequent points in time starting at $t = 0.2$ and separated by 2 time units. The solution converges to the form given by Eq. (17). Parameters as in Supplementary Figure 4.

In Supplementary Figure 5 the exact solution, Eq. (16), is evaluated for the same set of parameters. As predicted by Eq. (17), it converges to an equilibrium in which $f(x)$ exponentially increases for positive x and exponentially decreases at a faster rate for negative x. Thus all predictions derived from the recipe are borne out: the behaviour of $f(x,t)$ resulting from the press-perturbation at $x = 0$ is dominated by the response amplifying towards larger x, and there is a second response decaying towards smaller x. In passing, we note that a more detailed comparison with results from CAT would show that the method not only predicts the quantitative rates of attenuation and decay along the x axis correctly for this example, but also the speed of propagation of the fronts in Eq. (16) and the denominator in Eq. (17).

From this example problem it is not difficult to construct a corresponding problem with a spatially modulated solution. For any fixed wave number $q > 0$ of the modulation, simply go over to the new dependent variable $g(x,t) = \mathrm{e}^{\mathrm{i}qx} f(x,t)$. Expressed in terms of this new variable, the drift-diffusion-growth equation, Eq. (12), becomes

$$\frac{dg(x,t)}{dt} = rg(x,t) - v\left(\frac{d}{dx} - \mathrm{i}q\right) g(x,t) + D\left(\frac{d}{dx} - \mathrm{i}q\right)^2 g(x,y). \tag{23}$$

The responses of $g(x,t)$ to localised press perturbations, which can be computed directly from those of $f(x,t)$, are modulated patterns with wavelength $2\pi/q$.

It is instructive to compare the approach used here to the linear stability analysis conventionally performed in the study of pattern formation [25]. The conventional approach interprets $\hat{K}(\xi)$ as specifying, for real-valued ξ, the linear growth rate of sinusoidal modulations of $f(x,t)$ with wave number ξ, i.e. wavelength $2\pi/\xi$ [or as a corresponding eigenvalue of the linear operator given by the right-hand-side of Eq. (18)]. The unperturbed system, Eq. (12), is considered stable if $\mathrm{Re}[\hat{K}(\xi)] < 0$ for all real ξ, and unstable if $\mathrm{Re}[\hat{K}(\xi)] > 0$ for some ξ. In our example $\mathrm{Re}[\hat{K}(\xi)] = r - D\xi^2$. The system is therefore linearly stable for $r < 0$ and linearly unstable for $r > 0$ (because then $\mathrm{Re}[\hat{K}(0)] = r > 0$). The transition to instability occurs at $r = 0$, exactly when $\xi_+ \approx -r\mathrm{i}/v$ crosses the real axis, thus signalling the transition from attenuating

to amplifying system responses to localised press perturbations. We have thus shown that, for this example, where both approaches are applicable, the conventional stability analysis and the approach of Sec. 6.3 are equivalent.

A limitation of the method of Sec. 6.3 is that it works by constructing stable steady-state solutions. When responses to pulse perturbations do not drift sufficiently fast to the left or the right on the x axis, responses to press perturbations can pile up at one location and no stable steady-state solutions exist in linear models. Such systems called *absolutely unstable*. By contrast, the instability discussed in the previous paragraph is called a *convective instability*, because of any fix x a steady state is eventually reached. Our simple model, Eq. (12), becomes absolutely unstable for $4Dr \geq v^2$ [26]. Unless $v = 0$, the transition to absolute instability therefore always occurs after the transition to convective instability. This ordering of transitions is generic in extended 1D systems when the symmetry between left and right along the coordinate axis is broken. It permits us here to pin down transitions to convective instability while disregarding complications due to absolute instability.

Supplementary Note 7: Analytic theory for the formation of dome patterns

Mathematically, there are two routes that lead to amplification of top-down cascades with increasing primary production in the non-linear SSSM. The starting point for both is an increase in phytoplankton due to nutrient enrichment, and the resulting conventional, un-modulated bottom-up effect. Because this effect is amplifying towards larger body sizes, it does not only lead to an overall increase in community biomass (Figs. 1a and 3a of the main text), but also makes the fitted slope S of the size spectrum less steep (Fig. 3b). The response of the size spectrum modulation to these changes is a second-order phenomenon: it is a change in how the system responds to pressures resulting from other changes in the system. A rigorous analytic study of this phenomenon would require an extension of the linear SSSM by terms quadratic in $b(u)$, to be derived from the non-linear SSSM; and then a mathematical analysis of this model. Here a simpler, heuristic approach is chosen, where the two effects are described by modifying the formal description of the base state of the linear model itself, followed by an analysis of the consequences that these modifications have for linear responses.

7.1 Implications of a changing size-spectrum slope

The increase in the fitted slope S seen in simulations (Fig. 3b of the main text) is due to trophic amplification of bottom-up effects. In the linear theory, it is represented by the imaginary singleton zero of $\hat{K}(\xi)$ in Supplementary Figure 3. Mathematically, one expects that trophic amplification leads to small deviations of size spectra form a power-law form (CAT, Section 9.6). However, for the semi-quantitative considerations here it is fair to disregard this and

equate the fitted size-spectrum slope S with a modified power-law exponent $2 - \lambda$. By the same amount by which S increases in Fig. 3b, the dash-dotted line in Supplementary Figure 3 therefore effectively sinks. Applying the recipe given above for the interpretation this kind of graph to a situation where the dash-dotted line sinks as nutrients increase, one concludes that with increasing nutrient supply bottom-up cascades become (more) attenuating, top-down cascades become less attenuating and conventional bottom-up effects less amplifying.

The ecological interpretation of this is straightforward. An increase in $S \approx 2 - \lambda$ means that, in comparison with the biomass of smaller species, the biomass of larger species increases. The effects of responses to pressures that propagate to larger species are therefore attenuated in relative terms. Their effects get "drowned-out" in the high overall biomass of large species. Likewise, the attenuation of the top-down cascade becomes weaker because, if large species are relatively more abundant, changes in their biomass by a small proportion can lead to more pronounced proportional changes in the abundances of smaller species.

The amplification of top-down effects through an increase of the size-spectrum slope S is thus more a matter of book-keeping than of actual ecology, which results from considering relative rather than absolute changes in abundance in empirical data. Nevertheless it is important, because for practical reasons ecologists prefer to compare relative rather than absolute changes [19].

7.2 Implications of overall biomass increase

To understand the effect of an overall increase in community biomass on size-spectrum dynamics, a closer look at the interaction kernel $\tilde{K}(w)$ is required. In CAT (Eqs. [81], [86]), an explicit expression for $\tilde{K}(w)$ was derived in terms of its Fourier transform. With the minor modifications of the SSSM introduced in Supplementary Note 1 above, it reads

$$\hat{K}(\xi) = \tilde{B}_{\text{tot}}\hat{\kappa}_0(\xi + \nu\mathrm{i})\,\hat{\beta}(-\xi - \nu\mathrm{i})\hat{\beta}(\xi) + \overbrace{\hat{X}(\xi)}^{\text{Food-Web}}, \tag{24}$$

where (for the oligotrophic regime) $\nu = 1 - q$; the function $\hat{\kappa}_0$ describes individual-level interactions and is given by

$$\hat{\kappa}_0(\xi + \nu\mathrm{i}) = \overbrace{\frac{\alpha h^2 y\gamma}{(y\gamma\tilde{\phi} + h)^2}\hat{s}(\xi)}^{\text{Feeding}} - \overbrace{\frac{y\gamma h}{y\gamma\tilde{\phi} + h}\hat{s}(-\xi - \nu\mathrm{i})}^{\text{Predation}} + \overbrace{\frac{y^2\gamma^2\tilde{\mathcal{N}}h}{(y\gamma\tilde{\phi} + h)^2}\hat{s}(\xi)\hat{s}(-\xi - \nu\mathrm{i})}^{\text{Release}}; \tag{25}$$

$\hat{\beta}(\xi)$ is the Fourier transform of the population structure $\tilde{\beta}(u)$ given by Eq. (7) above, with the two factors in Eq. (24) representing the computation of the individual-based community size spectrum $\mathcal{N}(m)$ from the species size spectrum $B(m_*)$ (Supplementary Table 1) and the computation of species-level dynamics from individual-level interactions (Eq. (1)); and

$$\hat{X}(\xi) = \rho \left[\exp\left(-\frac{\sigma_r^2 \xi^2}{2} \right) - \exp\left(\frac{\sigma_r^2 (\lambda - 2)^2}{2} \right) \right]. \tag{26}$$

This last term describes the damping of short wavelength (i.e. large $|\operatorname{Re}\xi|$) modulations of the size spectrum due to food-web effects. It differs from the form used in CAT by having the constant $\exp\left(\sigma_r^2(\lambda-2)^2/2\right)$ ($= 1.003$ for parameters as in Supplementary Table 1) in place of 1.

The new parameter y in Eq. (25) equals $y = 1$ for the linear SSSM (but see below); $\tilde{\mathcal{N}}$ is the coefficient scaling the community size spectrum of the base state; $\tilde{\phi} = \tilde{\mathcal{N}}\, \hat{s}\big(i(\lambda-2)\big)$ scales food availability; and $\hat{s}(\xi)$ is the Fourier transform (on a logarithmic scale) of the predator-prey mass-ratio window $s(x)$. For our choice of $s(x)$ (Supplementary Table 1), this is

$$\hat{s}(\xi) = (2\pi)^{1/2} \sigma_s \exp\left[-\frac{\sigma_s^2 \xi^2}{2} \right] \beta^{-\mathrm{i}\xi}. \tag{27}$$

The three named terms in Eq. (25) correspond to different ecological effects. From the derivation of these terms in CAT (Sec. 6.3), one see that *Feeding* describes increases in population growth with increasing density of food; *Predation* describes population decline with increasing density of predators; and the last term, *Release*, describes release from predation in situations where consumers of the focal species are (partially) satiated [27]. The focal size class then experiences a safety-in-numbers effect that reduces its *per capita* predation mortality. This release from predation increases as the focal size class and species of similar size become more abundant, and *vice versa*.

We included a factor y in several places in Eq. (25) as a simple way to model the effects of an overall increase of community biomass in the SSSM. Values $y \neq 1$ describe the effects of scaling the biomass of the based state by a factor y, which formally results from multiplying $\tilde{B}_{\text{tot}}$ by y. This includes the direct effect *via* Eq. (25), and indirect effects through the scale factors for community size spectrum $\tilde{\mathcal{N}}$ and food availability $\tilde{\phi}$. Alternatively and perhaps more transparently, $y \neq 1$ can be interpreted as describing effective re-scaling of the value of the coefficient of search&attack rates γ: every occurrence of γ in Eq. (25) goes along with a factor y.

Important for the following is that changes in y affect the magnitude of the three named terms in $\hat{\kappa}_0$ in different ways. For y smaller than $h/\gamma\tilde{\phi}$, the main effect of increasing y is to enhancing the role of the term named Release compared the terms Feeding and Predation, because y enters quadratically in the numerator of Release but only linearly in the numerators of Feeding and Predation. This makes ecological sense, because Release describes an effect resulting from (partial) saturation of consumers. At low food abundance it plays no important role.

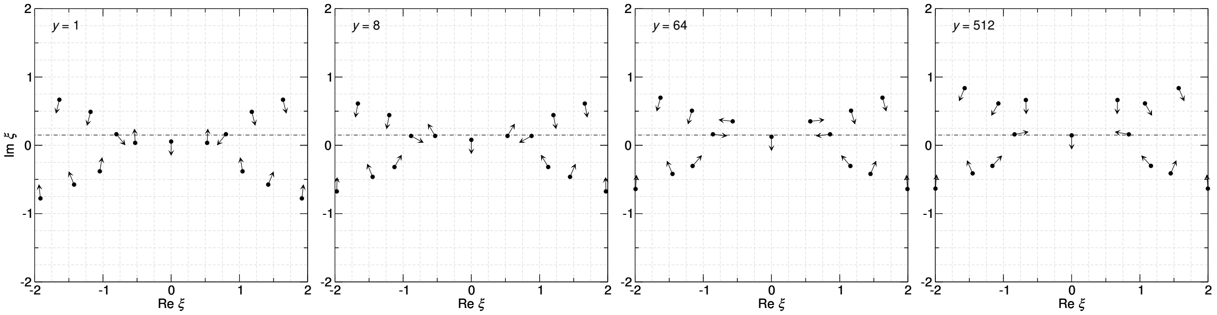

Supplementary Figure 6: Location of the zeros of $\hat{K}(\xi)$ in the complex plane and arrows indicating $\arg d\hat{K}(\xi)/d\xi$ for increasing enrichment y. For $y \approx 8$ the pair of zeros corresponding to top-down cascades crosses the dash-dotted line, signifying a transition from attenuating to amplifying top-down cascades.

However, as y increases in magnitude beyond $h/\gamma\tilde{\phi}$ and the expression $y\gamma\tilde{\phi} + h$ in the denominators in Eq. (25) can be approximated as $y\gamma\tilde{\phi}$, further increases in y lead to a decline of the importance of Feeding (which then scales as $1/y$) relative to the terms Predation and Release (where y effectively cancels out). Ecologically, this is the transition from density-dependent feeding at low food abundance to effectively density-independent feeding when food is plenty. Supporting this analysis, the expression for $\hat{\kappa}_0$ derived in CAT for the so-called eutrophic regime is identical to that obtained by taking the limit $y \to +\infty$ in Eq. (25).

With this preparation, it is now possible to study mathematically the effects of biomass scaling on how responses to press perturbation propagate along the size spectrum. All one needs to do is to see how the zeros of $\hat{K}(\xi)$ in the complex plane respond to changes in y. For the parameters used here (Supplementary Table 1), this is shown in Supplementary Figure 6.

Interestingly, the strongest response to changes in y in Supplementary Figure 6 is that of the pair of zeros corresponding to the top-down cascade. It moves upward ($\operatorname{Im}\xi$ increases) and crosses the line $\operatorname{Im}\xi = \lambda - 2$ at about $y = 8$. After crossing the line, the arrows attached to the zeros point away from the line, which means that, by to the rules explained above, the top-down cascade has become amplifying!

With further increases in y, the pair of zeros climbs further, signifying stronger amplification in the linear model (in the full model, non-linear effects set limits to this amplification), until about $y = 64$, where the arrows reverse orientation. Remarkably, biomass scaling has a much weaker effect on the positions of the other zeros, in particular those corresponding to bottom-up cascades.

Figure 6 thus demonstrates that, with the parameters used here, the linear SSSM predicts a transition from attenuating to amplifying top-down cascades when overall community biomass increases. The analytic formulation of the SSSM now allows us to answer in addition the

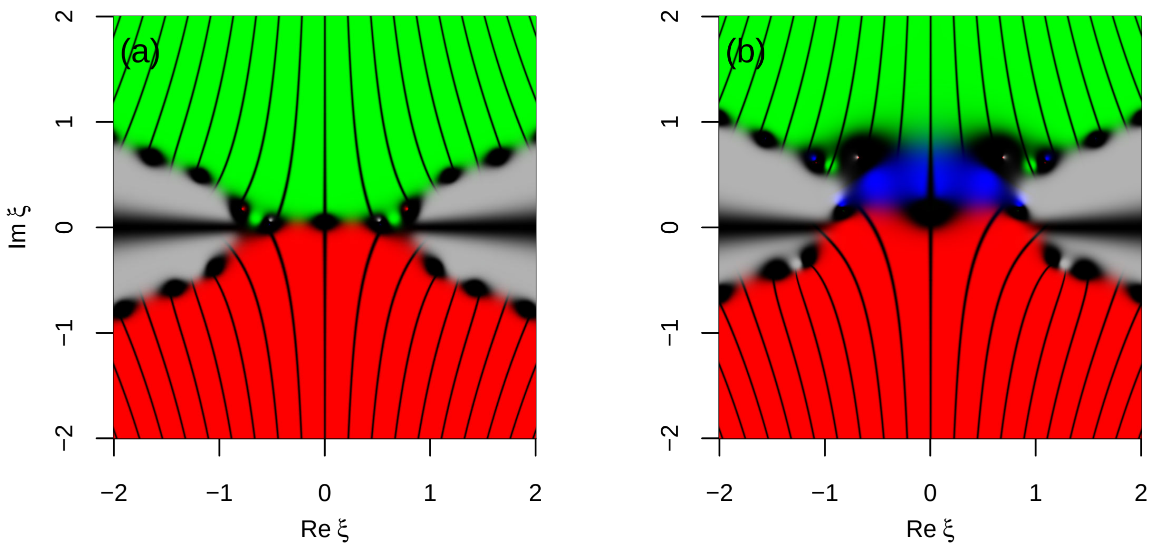

Supplementary Figure 7: Areas of dominance of terms contributing to $\hat{K}(\xi)$ (Eq. (24)) in the complex ξ-plane for (a) $y = 1$ and (b) $y = 512$. Colouring is green for Feeding, red for Predation, blue for Release, and grey for the Food-Web term. For each term Term(ξ), black lines indicate where the imaginary part of Term(ξ) is close to zero, thus providing information about how $\arg$ Term(ξ) changes through the complex plane. The plots were generated by setting the colour intensity for each term to (colour intensity) $= \exp[-7\,|1 - \text{Term}(\xi)/\hat{K}(\xi)|^3]\{1 - [\cos\arg\text{Term}(\xi)]^{100}\}$ and adding intensities. The small coloured spots in both panels are artefacts of this method.

following two questions: what is the underlying mechanism and how generic is it?

The key to addressing these two questions is the observation that in different parts of the complex plane, i.e. depending on ξ, the function $\hat{K}(\xi)$ is dominated by different terms (in the sense that contributions by all other terms are comparatively small). Figure 7a indicates by different colours the areas dominated by the three terms Feeding, Predation (both times the factor $\tilde{B}_{\text{tot}}\hat{\beta}(-\xi - \nu\text{i})\hat{\beta}(\xi)$ from Eq. (24)), and Food-Web for $y = 1$. Their relative positions follow from generic considerations and the formula for Fourier transforms, Eq. (11), with complex argument: Feeding dominates in the upper complex plane because it describes predominantly the dependence on smaller (food) species; Predation dominates in the lower complex plane because it describes the impacts of larger species; and Food-Web dominates towards the left and the right along the real axis because all other terms decline to zero in this direction (they must when the predator-prey size ratio window is continuous and bounded) while Food-Web contains a constant contribution (here $\rho\exp\left(-\sigma_r^2(\lambda - 2)^2/2\right)$), which describes reduced competition amongst species of very similar size but with otherwise different trophic roles, as explained in Supple-

mentary Note 1 above.

Except for the possibility of zeros due to the $\hat{\beta}$ factors in Eq. (24), which plays no role here, zeros of $\hat{K}(\xi)$ will typically arise only when two or more of the four terms Feeding, Predation, Release, and Food-Web cancel each other. A simple example illustrating this principles is the function $\sin(\xi) = 0.5 \exp(\mathrm{i}\xi) - 0.5 \exp(-\mathrm{i}\xi)$ for complex-valued arguments ξ. The term $0.5 \exp(\mathrm{i}\xi)$ dominates $\sin(\xi)$ in the lower half plane, and $0.5 \exp(-\mathrm{i}\xi)$ dominates in the upper half plane. Because $\exp(\mathrm{i}\xi)$ itself has no zeros in the complex plane, $\sin(\xi) = 0$ requires that the two contributions have the same magnitude (specifically, the same absolute value) and cancel each other. This is the case only along the real axis. In addition, for a zero to occur, $\arg[\exp(\mathrm{i}\xi)]$ and $\arg[\exp(-\mathrm{i}\xi)]$ must be identical. Indeed, this happens at certain points along the real axis, because $\arg[\exp(\mathrm{i}\xi)]$ and $\arg[\exp(-\mathrm{i}\xi)]$ depend differently on $\mathrm{Re}\,\xi$. This is why the zeros of $\sin(\xi)$ are lined up along the real axis. By the same reasoning, the zeros of $\hat{K}(\xi)$ will generally line the boundaries between areas of dominance of terms, where contributions from different ecological effects balance each other.

These considerations, combined with Supplementary Figure 7a, explain the positions of the zeros in Supplementary Figures 3 and 6 for $y = 1$. Specifically, the zeros corresponding to the conventional bottom-up and top-down effects result essentially form a balance of Feeding and Predation. The zero corresponding to the bottom-up cascade is already strongly affected by the Food-Web term; without food-web effects, the resulting perturbation response would be more strongly amplifying. All other zeros, representing strongly damped cascades, result from either a direct balance between Food-Web and Feeding or between Food-Web and Predation.

To understand why the conventional top-down effect responds particularly sensitively to biomass scaling, as illustrated in Supplementary Figure 6, it is useful to consider first the situation for the very high scaling factor $y = 512$. The dominance of terms in the complex plane for $y = 512$ is shown in Supplementary Figure 7b. As for $y = 1$, generic considerations are sufficient to understand the relative positions of areas of dominance. Predation dominates over Release in the lower complex plane, because Predation describes predominantly the impacts of larger species, while Release describes effective interactions amongst species of similar size. Release gradually replaces Feeding with increasing y because of the dependencies of these terms on y discussed above. However, higher up in the complex plane Feeding holds out longer with increasing y because it describes dependence on smaller species, rather than on species of similar size. Finally, dominance of Release develops first in areas with small $\mathrm{Re}\,\xi$ because this effect, involving a chain of two predator-prey interactions (prey-predator-prey), is less size specific than Feeding and Predation. It therefore contributes over a wider range in w to $\tilde{K}(w)$ and, as result, over a narrow range in $\mathrm{Re}\,\xi$ to $\hat{K}(\xi)$. Mathematically in Eq. (25), this narrow range results from the additional factor $\hat{s}(-\xi - \nu \mathrm{i})$ compared to Feeding, which is localised near $\mathrm{Re}\,\xi = 0$.

As Release, with increasing y, breaks up the boundary between the areas dominated by Feeding and Predation, the purely imaginary zero corresponding to the conventional bottom-up effect gets located on the boundary between Release and Predation, while the pair of zeros corresponding to the conventional top-down effect moves along with the boundary between Release and Feeding (Supplementary Figure 7). This, too, can be understood from generic considerations.

The imaginary singleton cannot be located between Release and Feeding, because the two terms have the same sign and cannot compensate each other for un-modulated perturbation responses (i.e. $\mathrm{Re}\,\xi = 0$). The balance must largely be between (positive) Release and (negative) Predation.

For the pair of zeros corresponding to the conventional top-down effect, the situation is opposite. Consider first a population located in a trough of a dome pattern for $y = 1$. The population's size is in equilibrium despite being comparatively low because of a balance of gains (Feeding term) by enhanced food availability from the dome where species are by an approximate factor β smaller and losses (Predation term) due to predation from the dome with species that are approximately β times larger (for focal species located in domes the effects are opposite). This balance is mathematically represented by the corresponding zero in the complex plane.

With $y = 512$, species in troughs experience strong additional losses due to comparatively low predation release (Release term). These losses cannot be compensated by additional losses from predation, only by gains from feeding. Hence Feeding and Release terms must balance in this case and the corresponding pair of zeros be located in the complex plane on the boundary between the areas dominated by these two terms. The pair of zeros therefore moves upward into the complex plane as the area of dominance of Release expands with increasing y.

Even before Release attains a dominating role, it skews the balance between Feeding and Predation in such a way that, with increasing y, the pair of zeros representing the conventional top-down cascade gradually moves upward in the complex plane. Correspondingly, top-down cascades in size spectra gradually become less attenuating with enrichment. Eventually, they become amplifying. *This is the explanation of the amplification of top-down cascades resulting from an overall increase in community biomass.* Based on this explanation, the phenomenon can be expected to be generic: model details did not play a role in explaining it. There is no corresponding mechanism leading to amplification of bottom-up cascades with enrichment.

In the specific case of our model, a comparison of the Feeding and Release terms in Eq. (25) shows that whenever $\arg \hat{s}(-\xi - \nu\mathrm{i}) = \pm\pi$ the arguments of the two terms differ by $\pm\pi$, implying that they can cancel each other. Using Eq. (27), this evaluates to a condition

$$\mathrm{Re}\,\xi = \pm\frac{\pi}{\ln\beta - \sigma_s^2\,(\nu + \mathrm{Im}\,\xi)}. \tag{28}$$

The top-down response becomes amplifying when the corresponding zeros cross the line $\operatorname{Im}\xi = \lambda - 2$ (disregarding changes in the size-spectrum slope). Putting this into Eq. (28) and making use of $\nu = 1 - q$ and $\lambda = 2 + q - n$, one can approximate the maturation body mass ratio separating successive local maxima of the resulting size-spectrum modulation as

$$\exp\left(\left|\frac{2\pi}{\operatorname{Re}\xi}\right|\right) = \beta^2 \exp[-2\,\sigma_s^2\,(1-n)]. \tag{29}$$

With parameters as in Supplementary Table 1, this evaluates to $81163 \approx 10^{4.9}$. Equation (29) thus provides a good approximation of the distance between domes seen in simulations of the non-linear SSSM, reported to be in the range 10^4 to 10^5 in Fig. 3d of the main text. This agreement further confirms the validity of the analytic theory.

It is noteworthy that, as see from Supplementary Figure 7, both the zero corresponding to the conventional bottom-up effect and the zeros corresponding to the top-down cascade are determined by the balance between the three terms Feeding, Predation, and Release in the expression for $\hat{\kappa}_0$ given by Eq. (25). That is, the relevant zeros of $\hat{K}(\xi)$ are well approximated the by zeros of $\hat{\kappa}_0(\xi + \nu\mathrm{i})$. But the function $\hat{\kappa}_0$ describes exclusively individual-level phenomena without regard to species ID—the life history parameters x_0 and η do not enter it. An implication of this observation is that details of life-history are unlikely to play an important role in dome formation.

7.3 Changes in the direction of propagation of trophic cascades in the linear SSSM

In this section, we briefly consider a questions that arise from the analytic theory above and must be addressed for completeness: What is the ecological meaning of the change in orientation at large y of the arrows in Supplementary Figure 6 for the pair of the zeros corresponding to top-down cascades?

In the linear SSSM, modified by inclusion of the parameter y in Eq. (25), the rise in the complex plane with increasing y of the pair of zeros of $\hat{K}(\xi)$ corresponding to top-down cascades is accompanied by a slow rotation of the attached arrow (Supplementary Figure 6). At $y \approx 64$ the arrow turns to point down, rather up, towards the line $\operatorname{Im}\xi = \lambda - 2$. The linear theory predicts that in this case perturbation responses will propagate towards species larger than those perturbed and attenuate with larger size ratios.

In principle, this is ecologically plausible. Above it was explained that, for large y, this pair of zeros results from a balance between Feeding and Release. Considering that neither term describes an effect resulting from species that are much larger than the focal species (as Predation does), but the Feeding term depends on smaller species, a propagation of the perturbation response towards larger species can be expected. Attenuation of this bottom-up response is a plausible expectation, because, for nearly satiated consumers, changes in the abundance of the

prey of a focal size class have only little effect on Feeding, so that only small changes in the abundance of the focal size class are required to compensated this through Release (i.e. release from even higher predation). From these considerations, one would hence expect dome patterns to disappear with strong enrichment.

In simulations of the non-linear SSSM, however, this is not observed (Fig. 3c of the main text). The reason is probably that, with large eutrophication parameter y, system states differ too much from the underlying base state to approximate their dynamics by the linear SSSM. For example, troughs in species size spectra of highly eutrophic systems might be so deep that species with sizes located within domes experience food limitation, despite the high overall abundance of biomass. This would undermine the chain of reasoning above. In situations as the present, where non-linear and linear SSSM disagree in their predictions, the full, non-linear model should be considered the more reliable. The expectation that domes might disappear with strong enrichment thus seems to be an artefact of the linearization.

7.4 Conclusions

This concludes our mathematical analysis of the mechanisms leading to dome formation in the SSSM. Enrichment can transform attenuating top-down cascades into amplifying cascades by two mechanisms. Firstly, through an increase of the fitted size-spectrum slope S, which implies a sinking of the dash-dotted line in Supplementary Figure 3. This mechanism has the opposite (damping) effect on bottom-up cascades. Secondly, enrichment amplifies top-down cascades by an overall increase in community biomass, which lets the pair of zeros corresponding to top-down cascades in Supplementary Figure 3 rise. The latter is caused by (partial) satiation of predators, which reduces their ability to control the abundance of their prey because (1) their feeding rate becomes less dependent on prey abundance and (2) the prey of satiated consumers experiences a safety-in-numbers effect [27]. Equilibrium prey abundances therefore respond stronger to changes in predator abundance, which enhances top-down cascades. (There is no corresponding effect for bottom-up cascades.)

With a sinking dash-dotted line and a rising pair of zeros for top-down cascades, the two will eventually cross, signalling the transition to amplifying top-down cascades. While our analytic approach is too coarse to compute for exactly what value of enrichment x this will happen, we can give an upper bound. For any value of $x > 1$, the resulting increase in overall biomass is at least x-fold (in fact, it is larger because of trophic amplification). For $x > 8$, the corresponding value of y must therefore be > 8 as well. But for any y just slightly larger than 8 the relevant pair of zeros will have crossed the dash-dotted line (Supplementary Figure 6), especially if that line has sunken below the base-state level. Hence, amplifying top-down cascades arise in the non-linear SSSM at some value of x between 1 and 8. This is the range within which dome formation is seen in simulations. We conclude that amplification of top-down cascades with

enrichment is causing dome formation. The conclusion is supported by the observation that the mass ratio separating consecutive domes in the nonlinear SSSM (10^D, with D as in Fig. 3d) is numerically close to the predicted mass ratio separating the maxima of top-down cascades at the onset of amplification, given by Eq. (29).

Supplementary References

[1] Rossberg AG (2012) A complete analytic theory for structure and dynamics of populations and communities spanning wide ranges in body size. *Adv. Ecol. Res.* 46:429–522.

[2] Hartvig M, Andersen KH, Beyer JE (2011) Food web framework for size-structured populations. *J. Theor. Biol.* 272(1):113–122.

[3] Rossberg AG, Farnsworth KD (2011) Simplification of structured population dynamics in complex ecological communities. *Theor. Ecol.* 4(4):449–465.

[4] Fisher RA (1930) *The Genetical Theory of Natural Selection.* (Oxford University Press, Oxford).

[5] van den Bosch F, Metz JAJ, Diekmann O (1990) The velocity of spatial population expansion. *J. Math. Biol.* 28(5):529–565.

[6] Rossberg AG (2013) *Food Webs and Biodiversity: Foundations, Models, Data.* (Wiley).

[7] Naisbit RE, Rohr RP, Rossberg AG, Kehrli P, Bersier LF (2012) Phylogeny versus body size as determinants of food-web structure. *Proc. R. Soc. B* 279(1741):3291–3297.

[8] Kiørboe T, Hirst AG (2014) Shifts in mass scaling of respiration, feeding, and growth rates across life-form transitions in marine pelagic organisms. *Am. Nat.* 183(4):E118–E130.

[9] Barnes C, Maxwell D, Reuman DC, Jennings S (2010) Global patterns in predator-prey size relationships reveal size dependency of trophic transfer efficiency. *Ecology* 91(1):222–232.

[10] Hansen B, Bjornsen PK, Hansen PJ (1994) The size ratio between planktonic predators and their prey. *Limnol. Oceanogr.* 39(2):395–403.

[11] Lampert W (1987) Laboratory studies on zooplankton-cyanobacteria interactions. *N. Z. J. Mar. Freshw. Res.* 21(3):483–490.

[12] Rodriguez J, Mullin MM (1986) Relation between biomass and body weight of plankton in a steady state oceanic ecosystem. *Limnol. Oceanogr.* 31:361–370.

[13] Quiñones RA, Platt T, Rodriguez J (2003) Patterns of biomass-size spectra from oligotrophic waters of the Northwest Atlantic. *Prog. Oceanogr.* 57:405–427.

[14] Rossberg AG, Farnsworth KD, Satoh K, Pinnegar JK (2011) Universal power-law diet partitioning by marine fish and squid with surprising stability-diversity implications. *Proceeding R. Soc. B* 278(1712):1617–1625.

[15] Hendriks AJ, Mulder C (2008) Scaling of offspring number and mass to plant and animal size: Model and meta-analysis. *Oecologia* 155(4):705–716.

[16] Hindmarsh AC, et al. (2005) SUNDIALS: Suite of nonlinear and differential/algebraic equation solvers. *ACM Trans Math Soft* 31(3):363–396.

[17] R Core Team (2017) *R: A Language and Environment for Statistical Computing*. (R Foundation for Statistical Computing, Vienna, Austria).

[18] Boccara N (1990) *Functional Analysis: An Introduction for Physicists*. (Academic Press, New York).

[19] Shurin JB, et al. (2002) A cross-ecosystem comparison of the strength of trophic cascades. *Ecol. Lett.* 5(6):785–791.

[20] Boudreau PR, Dickie LM, Kerr SR (1991) Body-size spectra of production and biomass as system-level indicators of ecological dynamics. *J. Theor. Biol.* 152(3):329–339.

[21] Thiebaux ML, Dickie LM (1992) Models of aquatic biomass size spectra and the common structure of their solutions. *J. Theor. Biol.* 159(2):147–161.

[22] Thiebaux ML, Dickie LM (1993) Structure of the body-size spectrum of the biomass in aquatic ecosystems: A consequence of allometry in predator-prey interactions. *Can. J. Fish. Aquat. Sci.* 50:1308–1317.

[23] Abramowitz M, Stegun IA, eds. (1972) *Handbook of Mathematical Functions*. (Dover, New York).

[24] Sheldon RW, Prakash A, Sutcliffe, Jr. WH (1972) The size distribution of particles in the ocean. *Limnol. Oceanogr.* 17:327–340.

[25] Cross MC, Hohenberg PC (1993) Pattern formation outside of equilibrium. *Rev Mod Phys* 65:851.

[26] Cross MC (1988) Structure of nonlinear traveling-wave states in finite geometries. *Phys. Rev. A* 38(7):3593–3600.

[27] Karban R (1982) Increased reproductive success at high densities and predator satiation for periodical cicadas. *Ecology* 63(2):321–328.
