# Supplementary material for: Dome patterns in pelagic size spectra reveal strong trophic cascades
Source: Nat Commun. 2019 Sep 27;10:4396. doi: 10.1038/s41467-019-12289-0 (PMC6764997; doi:10.1038/s41467-019-12289-0)
Supplement: Supplementary file 3 — Reporting Summary [file 41467_2019_12289_MOESM3_ESM.pdf]

nature research

Corresponding author(s): Axel G. Rossberg

Life Sciences Reporting Summary

Nature Research wishes to improve the reproducibility of the work that we publish. This form is intended for publication with all accepted life science papers and provides structure for consistency and transparency in reporting. Every life science submission will use this form; some list items might not apply to an individual manuscript, but all fields must be completed for clarity.

For further information on the points included in this form, see Reporting Life Sciences Research. For further information on Nature Research policies, including our data availability policy, see Authors & Referees and the Editorial Policy Checklist.

Please do not complete any field with "not applicable" or n/a. Refer to the help text for what text to use if an item is not relevant to your study. For final submission: please carefully check your responses for accuracy; you will not be able to make changes later.

Experimental design

1. Sample size

Describe how sample size was determined.	This study did not generate new empirical data. The data we used was collected from the literature or supplied in numerical form by the respective study authors. The availability of suitable size-spectrum data sets in the literature therefore determined our sample size.

2. Data exclusions

Describe any data exclusions.	Data for "Wurmsee", "Binnenmuetzel", "Nehmitzsee", and "Tiefer See" by Tittel et al. (1998) and for the northern Laguna Iberá by Cózar et al. (2003) was excluded, because it is not averaged through time, despite high seasonal variability. From historical, repeated measurements of the size spectrum of Lake Superior only the last, most accurate one was used. The spectrum of La Caldera by was excluded Echevarría et al. (J. of Plankton Research 12(2), 41S-422, 1990) because of acknowledged potential uneven sampling of size classes.

3. Replication

Describe the measures taken to verify the reproducibility of the experimental findings.	Since this study is based on literature data, no independent replication was possible. Reproducibility is determined only based on our statistical analyses of the data. However, when discussing trends in the four size-spectrum characteristics, we provide in the article, for each of the characteristics except for the distance between domes D (where the trends is weak), references to work where similar trends have been observed.

4. Randomization

Describe how samples/organisms/participants were allocated into experimental groups.	We acknowledge in the article that covariates are likely to contribute to the observed scatter of data. We did not control for covariates because the required data is often not available.

5. Blinding

Describe whether the investigators were blinded to group allocation during data collection and/or analysis.	Because of the small number of data sets available and the challenges involved in identifying, assessing and extracting data sets and transforming them to a standard representation, we decided to perform all this work collaboratively rather than tasking blinded individuals.

Note: all in vivo studies must report how sample size was determined and whether blinding and randomization were used.

6. Statistical parameters

For all figures and tables that use statistical methods, confirm that the following items are present in relevant figure legends (or in the Methods section if additional space is needed).

n/a	Confirmed	
☐	☒	The exact sample size (*n*) for each experimental group/condition, given as a discrete number and unit of measurement (animals, litters, cultures, etc.)
☐	☒	A description of how samples were collected, noting whether measurements were taken from distinct samples or whether the same sample was measured repeatedly
☒	☐	A statement indicating how many times each experiment was replicated
☐	☒	The statistical test(s) used and whether they are one- or two-sided<br>*Only common tests should be described solely by name; describe more complex techniques in the Methods section.*
☒	☐	A description of any assumptions or corrections, such as an adjustment for multiple comparisons
☐	☒	Test values indicating whether an effect is present<br>*Provide confidence intervals or give results of significance tests (e.g. P values) as exact values whenever appropriate and with effect sizes noted.*
☐	☒	A clear description of statistics including central tendency (e.g. median, mean) and variation (e.g. standard deviation, interquartile range)
☐	☒	Clearly defined error bars in all relevant figure captions (with explicit mention of central tendency and variation)

See the web collection on statistics for biologists for further resources and guidance.

Software

Policy information about availability of computer code

7. Software

Describe the software used to analyze the data in this study.	For the data plotting and analyses, we used scripts coded in R (v. 3.4.2). The simulations were carried out using a flexible but complicated software, written by one of us in C++, that allows us to run, compare and analyze different kinds of size-spectrum models, including the model described in this manuscript.

For manuscripts utilizing custom algorithms or software that are central to the paper but not yet described in the published literature, software must be made available to editors and reviewers upon request. We strongly encourage code deposition in a community repository (e.g. GitHub). *Nature Methods* guidance for providing algorithms and software for publication provides further information on this topic.

Materials and reagents

Policy information about availability of materials

8. Materials availability

Indicate whether there are restrictions on availability of unique materials or if these materials are only available for distribution by a third party.	No materials were used in this study.

9. Antibodies

Describe the antibodies used and how they were validated for use in the system under study (i.e. assay and species).	No antibodies were used in this study.

10. Eukaryotic cell lines

a. State the source of each eukaryotic cell line used.	No cell lines were used in this study.
b. Describe the method of cell line authentication used.	No cell lines were used in this study.
c. Report whether the cell lines were tested for mycoplasma contamination.	No cell lines were used in this study.
d. If any of the cell lines used are listed in the database of commonly misidentified cell lines maintained by ICLAC, provide a scientific rationale for their use.	No cell lines were used in this study.

Animals and human research participants

Policy information about studies involving animals; when reporting animal research, follow the ARRIVE guidelines

11. Description of research animals

Provide all relevant details on animals and/or animal-derived materials used in the study.	No animals were used in this study.

Policy information about studies involving human research participants

12. Description of human research participants

Describe the covariate-relevant population characteristics of the human research participants.

This study did not involved human research participants.
