# Supplementary material for: Dome patterns in pelagic size spectra reveal strong trophic cascades
Source: Nat Commun. 2019 Sep 27;10:4396. doi: 10.1038/s41467-019-12289-0 (PMC6764997; doi:10.1038/s41467-019-12289-0)
Supplement: Supplementary file 4 — Description of Additional Supplementary Files [file 41467_2019_12289_MOESM4_ESM.pdf]

Description of Additional Supplementary Files

File Name: Supplementary Data 1
Description: Empirical size spectrum data analysed in this study

File Name: Supplementary Software 1
Description: Source code and a pre-compiled binary of the implementation of the non-linear Species Size Spectrum Model used in this study
